# *DEFECTIVE EMBRYO AND MERISTEMS* genes are required for cell division and gamete viability in *Arabidopsis*

**DOI:** 10.1371/journal.pgen.1009561

**Published:** 2021-05-17

**Authors:** Chin Hong Lee, Nathaniel P. Hawker, Jonathan R. Peters, Thierry G. A. Lonhienne, Nial R. Gursanscky, Louisa Matthew, Christopher A. Brosnan, Christopher W. G. Mann, Laurence Cromer, Christelle Taochy, Quy A. Ngo, Venkatesan Sundaresan, Peer M. Schenk, Bostjan Kobe, Filipe Borges, Raphael Mercier, John L. Bowman, Bernard J. Carroll

**Affiliations:** 1 School of Chemistry and Molecular Biosciences, The University of Queensland, St. Lucia, Australia; 2 Section of Plant Biology, One Shields Avenue, University of California at Davis, Davis, California, United States of America; 3 Institut Jean-Pierre Bourgin, INRAE, AgroParisTech, Université Paris-Saclay, Versailles, France; 4 School of Agriculture and Food Sciences, The University of Queensland, St. Lucia, Australia; 5 Institute for Molecular Bioscience and Australian Infectious Diseases Research Centre, The University of Queensland, St. Lucia, Australia; 6 Department of Chromosome Biology, Max Planck Institute for Plant Breeding Research, Cologne, Germany; 7 School of Biological Sciences, Monash University, Clayton Campus, Clayton, Victoria, Australia; Cornell University, UNITED STATES

## Abstract

The *DEFECTIVE EMBRYO AND MERISTEMS 1 (DEM1)* gene encodes a protein of unknown biochemical function required for meristem formation and seedling development in tomato, but it was unclear whether DEM1’s primary role was in cell division or alternatively, in defining the identity of meristematic cells. Genome sequence analysis indicates that flowering plants possess at least two *DEM* genes. *Arabidopsis* has two *DEM* genes, *DEM1* and *DEM2*, which we show are expressed in developing embryos and meristems in a punctate pattern that is typical of genes involved in cell division. Homozygous *dem1 dem2* double mutants were not recovered, and plants carrying a single functional *DEM1* allele and no functional copies of *DEM2*, i.e. *DEM1/dem1 dem2/dem2* plants, exhibit normal development through to the time of flowering but during male reproductive development, chromosomes fail to align on the metaphase plate at meiosis II and result in abnormal numbers of daughter cells following meiosis. Additionally, these plants show defects in both pollen and embryo sac development, and produce defective male and female gametes. In contrast, *dem1/dem1 DEM2/dem2* plants showed normal levels of fertility, indicating that *DEM2* plays a more important role than *DEM1* in gamete viability. The increased importance of *DEM2* in gamete viability correlated with higher mRNA levels of *DEM2* compared to *DEM1* in most tissues examined and particularly in the vegetative shoot apex, developing siliques, pollen and sperm. We also demonstrate that gamete viability depends not only on the number of functional *DEM* alleles inherited following meiosis, but also on the number of functional *DEM* alleles in the parent plant that undergoes meiosis. Furthermore, DEM1 interacts with RAS-RELATED NUCLEAR PROTEIN 1 (RAN1) in yeast two-hybrid and pull-down binding assays, and we show that fluorescent proteins fused to DEM1 and RAN1 co-localize transiently during male meiosis and pollen development. In eukaryotes, RAN is a highly conserved GTPase that plays key roles in cell cycle progression, spindle assembly during cell division, reformation of the nuclear envelope following cell division, and nucleocytoplasmic transport. Our results demonstrate that DEM proteins play an essential role in cell division in plants, most likely through an interaction with RAN1.

## Author summary

Up to half of the genes predicted from genome projects lack a known biological and biochemical function. Many of these genes are likely to play essential roles but it is difficult to reveal their function because minor changes in the genetic sequence can result in lethality and genetic redundancy can obscure analysis. Genome projects predict that flowering plants have at least two *DEM* genes that encode a protein of unknown cellular and biochemical function. In this paper, we use multiple combinations of *dem* mutants in *Arabidopsis* to show that *DEM* genes are essential for cell division and gamete viability. Interestingly, gamete viability depends not only on the number of functional copies of *DEM* genes in the gametes, but also on the number of functional copies of *DEM* genes in the parent plant that produces the gametes. We also show that DEM proteins interact with RAN, a highly conserved protein that controls cell division in all eukaryotic organisms.

## Introduction

The life cycle of plants involves the alternation between a diploid sporophytic phase and a haploid gametophytic phase. In flowering plants, the sporophyte is the dominant form representing the adult stage of the life cycle [1]. Cell divisions that generate the sporophytic adult plant, including the flowers and reproductive tissue, are restricted to regions termed meristems [2]. During the reproductive phase, specialized cells within floral organs undergo meiosis to generate haploid spores [3,4]. These haploid spores undergo two or three rounds of mitosis to generate multicellular gametophytes, namely pollen (microgametophytes) and embryo sacs (megagametophytes), but only one cell within the female and male gametophytes contribute to the formation of the zygote during sexual reproduction. While the sporophytes and gametophytes differ vastly in size and patterns of gene expression, they share a requirement for cell division and regulation of the cell cycle. However, mutations that disrupt cell division are often more severe in meiosis and post-meiotic mitosis during gametophyte development in flowering plants [5,6].

Pollen are derived from microspore mother cells that undergo meiosis to generate tetrads of four haploid microspores in a process known as microsporogenesis. Then during microgametogenesis, each microspore undergoes two additional rounds of mitosis to generate mature pollen. The first round of mitosis during microgametogenesis is asymmetric and gives rise to a smaller generative cell enclosed within the larger vegetative cell of pollen. The vegetative cell ceases to undergo further cell division, whereas the generative cell subsequently undergoes a second round of mitosis, which results in the production of two sperm cells contained within the vegetative cell cytoplasm [3,7].

Each embryo sac is derived from a megaspore mother cell, which undergoes meiosis to produce a functional megaspore. However, only one of the four meiotic products gives rise to a functional megaspore, and the other three products of meiosis undergo programmed cell death and degenerate [4]. During megagametogenesis, the functional megaspore undergoes three rounds of mitosis followed by cellularization and differentiation to give rise to a seven-cell embryo sac consisting of an egg cell, two synergid cells, a di-haploid central cell and three antipodal cells. The di-haploid central cell of the megagametophyte is formed by the fusion of two haploid cells during cellularization [8]. Sporophytic tissue surrounds the megagametophyte in a structure termed the ovule. In *Arabidopsis*, there are multiple ovules contained within each carpel of the flower. After fertilization, each ovule develops into a seed [9].

In dicot plant species, such as tomato and *Arabidopsis*, there are two *DEFECTIVE EMBRYO AND MERISTEMS* (*DEM*) genes, which encode 72 kDa proteins of unknown biochemical function [10]. The *DEM1* gene was first identified in tomato, where it is required for organized cell division in the shoot and root meristems [10]. In tomato, *DEM1* is highly expressed in a punctate fashion in all meristematic and differentiating regions of the plant, and homozygous *dem1* mutants exhibit a loss of the shoot apical meristem, disorganized arrangement of cell patterning in the root meristem and a seedling lethal phenotype [10]. However, it remained to be demonstrated whether DEM1’s primary role was in cell division or alternatively, in defining the identity of meristematic cells in plants.

Here, we show that *DEM* genes have an essential role in cell division in *Arabidopsis*. Furthermore, DEM interacts with RAS-RELATED NUCLEAR PROTEIN 1 (RAN1) in a yeast two-hybrid system and in pull-down binding assays, and fluorescent proteins fused to DEM1 and RAN1 co-localize transiently during meiosis and pollen development.

## Results

### *DEM* gene family and distantly related homologues

**Fig 1:**
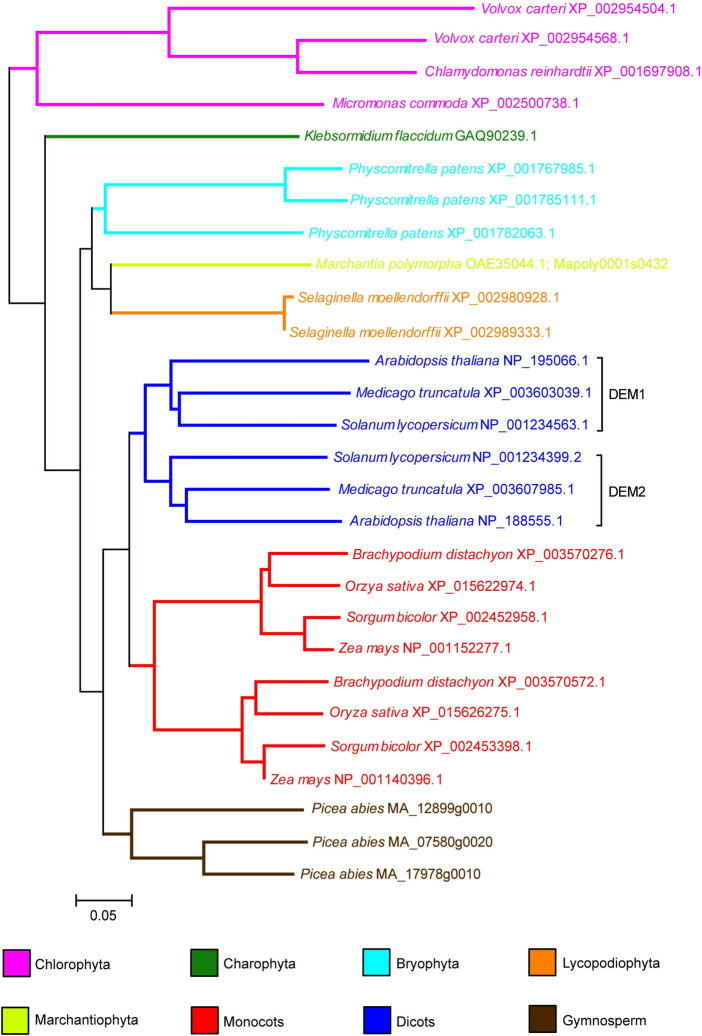
Phylogenetic tree of plant DEM proteins, rooted with the Chlorophyta sequences. Software and parameters used in the analysis are described in Materials and Methods. The name of the organism is indicated on the tree, followed by the accession number or the Conifer Genome Integrative Explorer gene ID for *Picea abies*. Scale bar indicates 0.05 substitutions per site.

*DEM1* and *DEM2* are located on chromosome 4 and 2 of *Arabidopsis*, respectively, and encode proteins that are 63% identical and 79% similar. A phylogenetic tree constructed from an alignment of predicted plant DEM proteins largely mirrors the accepted phylogeny of plants (Fig 1). A gene duplication event in a common ancestor of extant eudicot species formed the DEM1 and DEM2 clades (Fig 1). An independent gene duplication event also occurred in the lineage leading to extant grasses (Fig 1). Similarly, the multiple paralogs of DEM in *Physcomitrella* (mosses) are more closely related to each other than to DEM proteins in flowering plants (Fig 1), indicating that the common ancestor of land plants possessed a single DEM homologue.

Database searches revealed the presence of protein sequences with significant similarity to DEM in a wide range of divergent eukaryota, including Protista, Rhodophyta (red algae) and fungi (S1 and S2 Figs). However, DEM-like sequences were not found in the metazoan species analysed except for *Rhagoletis zephyria* and *Papilio xuthus*, which belong to the Diptera and Lepidoptera orders, respectively, in the class Insecta (S1 and S2 Figs). Otherwise, most of the DEM protein sequences cluster into their respective taxonomic groups (S1 Fig). These results suggest that the last common ancestor of eukaryotes possessed a *DEM* gene that has been retained in many lineages but lost in the majority of the metazoans.

Plant DEM proteins are highly conserved throughout their entire length, but particularly in the C-terminal half of the proteins (S2 Fig), and DEM proteins usually contain a predicted N-terminal myristoylation site (S1 Table). Analysis of plant DEM proteins using InterPro [11] showed sequence similarity to the yeast Vacuolar Import and Degradation 27 (VID27) protein [12,13], which contains a predicted WD40/YVTN repeat-like domain. In addition, the area of high conservation between DEM and VID27 protein includes a region that functions as a nuclear rim localization domain in *Schizosaccharomyces pombe* (accession AB027933) (S3 Fig) [14].

### *DEM* genes are expressed in tissues undergoing cell division

Previous studies using micro-arrays and RNA-seq have shown that both *Arabidopsis DEM* genes are predominantly expressed in differentiating tissue, but with *DEM1* showing highest expression in the early-stage embryos, vegetative shoot apices, developing roots and floral buds, and *DEM2* showing highest expression in developing seeds and pollen [15–17].

**Fig 2:**
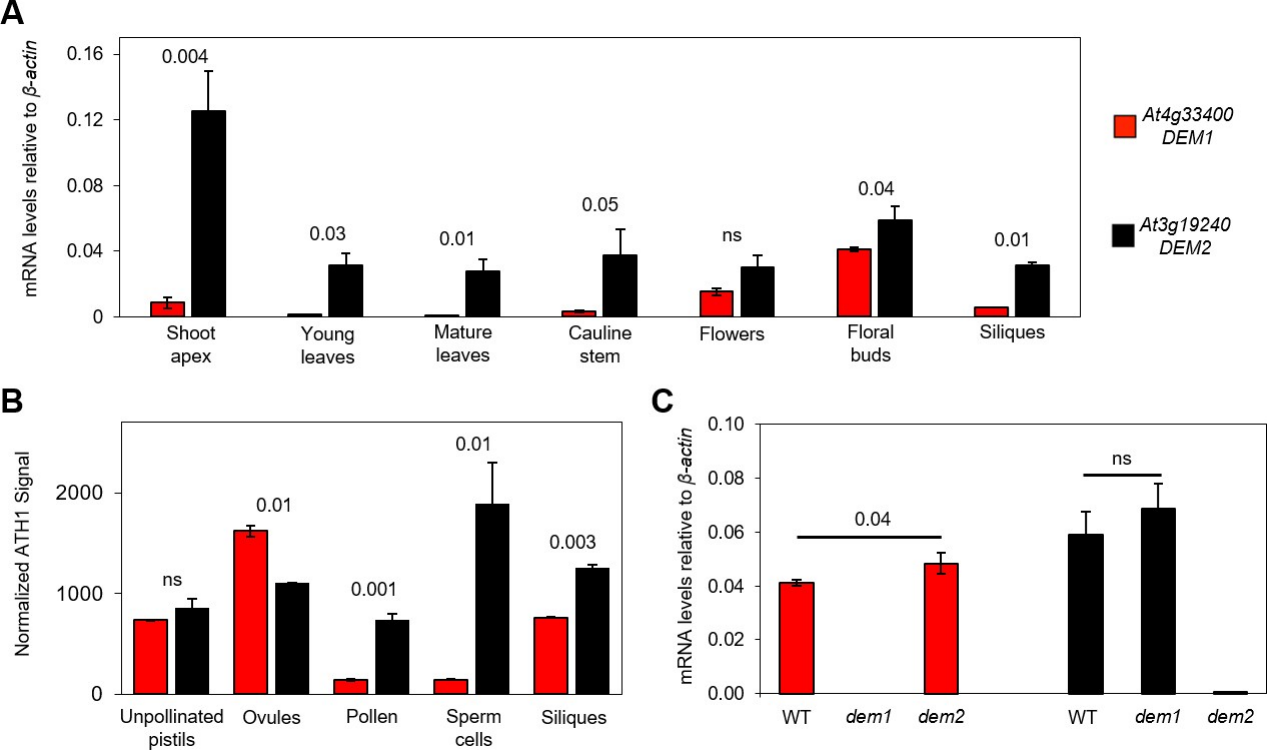
*DEM1* and *DEM2* mRNA levels in various tissues and sperm cells during vegetative and reproductive development. **(A)** Quantitative real-time reverse transcriptase PCR (qRT-PCR) was used to examine *DEM1* and *DEM2* mRNA levels in total RNA extracted from a range of *Arabidopsis* tissues. Ratios of mRNA levels to that of *β-actin* mRNA are shown (average ± S.E. for three biological replicates). **(B)** Microarray-based expression analysis of *DEM1* and *DEM2* in *Arabidopsis* reproductive tissues and FACS-purified sperm cells. The figure is based on raw data published by Pina *et al*. [18], Borges *et al*. [19] and Boavida *et al*. [20], and shows mean signal intensities ± S.E. from ATH1 Genechips normalized by the invariant set method, as previously described [19]. Data were derived from two biological replicates for siliques [18], microdissected ovules and unpollinated pistils (UPs) [20], and three biological replicates for pollen and FACS-purified sperm cells [19]. **(C)** qRT-PCR analysis of *DEM1* and *DEM2* mRNA levels in total RNA extracted from *Arabidopsis* wild-type (WT), *dem1-2* (*dem1*) and *dem2-2* (*dem2*) floral buds. Ratios of mRNA levels to that of *β-actin* mRNA are shown (average ± S.E. for three to six biological replicates). *P*-values were calculated using one-way ANOVA to indicate significant differences between *DEM1* and *DEM2* expression in the different tissues and sperm cells of wild-type plants (**A, B**), or between floral buds of wild-type and *dem* plants (**C**).

We used quantitative real-time reverse transcriptase PCR (qRT-PCR) to compare *DEM1* and *DEM2* mRNA levels across a range of tissues in *Arabidopsis*, and *DEM2* mRNA levels were higher than *DEM1* mRNA levels in all *Arabidopsis* tissues examined, although the differences weren’t significant in flowers (Fig 2A). *DEM2* mRNA levels were highest in the vegetative shoot apex and floral buds but was detectable in all tissues tested, whereas *DEM1* mRNA levels were highest in floral buds but also detectable in flowers, the vegetative shoot apex, siliques and cauline leaves (Fig 2A). Using published microarray data [18–20], we also compared the levels of *DEM1* and *DEM2* mRNA in reproductive tissues and FACS-purified sperm cells. This analysis showed that *DEM2* mRNA levels were much higher than *DEM1* mRNA levels in siliques, pollen and sperm cells (Fig 2B). *DEM1* and *DEM2* mRNA levels in microdissected ovules and unpollinated pistils were more comparable, although significantly higher *DEM1* mRNA levels were detected in microdissected ovules (Fig 2B).

**Fig 3:**
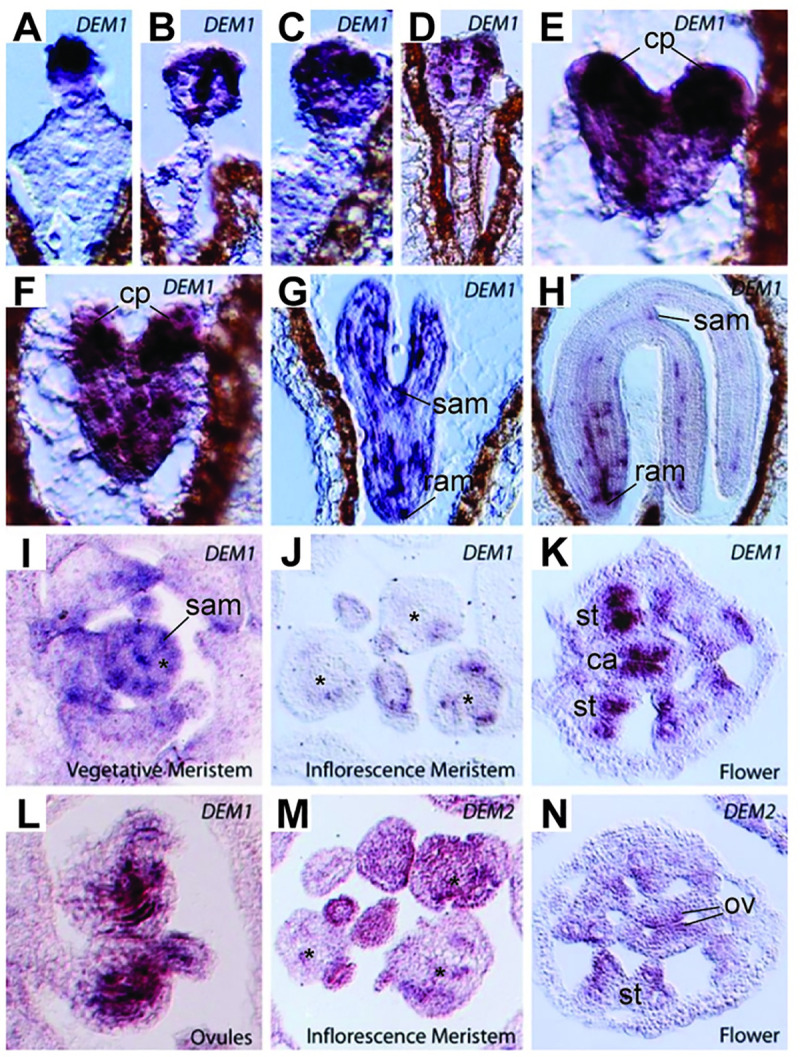
*DEM1* and *DEM2* mRNA expression detected by *in situ* hybridization in developing embryos and post-embryonic shoot tissues of *Arabidopsis* ecotype Ws-0. **(A-H)** Punctate expression of *DEM1* in developing embryos. **(A-D)** Globular stage. **(E-F)** Heart stage. **(G)** Torpedo stage. **(H)** Maturing embryo. **(I-J)** *DEM1* expression in shoot apices is not as clearly punctate as in developing embryos but is clearly expressed in vegetative shoot apical meristem and in some dividing cells. **(I)** Vegetative meristem. **(J)** Inflorescence meristem. **(K-L)** *DEM1* expression can clearly be seen in sporophytic tissues of developing anthers and developing ovules. **(K)** Flower. **(L)** Ovules. **(M-N)** *DEM2* is expressed in dividing cells of the inflorescence. **(M)** Inflorescence meristem. **(N)** Flower. cp, cotyledon primordia; ram, root apical meristem; sam, shoot apical meristem; *, central zone of meristem; st, stamen primordia; ca, carpel primordia; ov, developing ovules.

We also used *in situ* hybridization to gain a more precise understanding of the expression patterns of *DEM1* and *DEM2* in differentiating tissues of *Arabidopsis*. Both genes showed a punctate expression pattern that was most obvious for *DEM1* in developing embryos, meristems and differentiating tissues (Fig 3). During early stages of embryogenesis, a strong punctate expression pattern of *DEM1* is distributed throughout the embryo, but particularly in cotyledon primordia (Fig 3A–3F). At later stages of embryogenesis, *DEM1* expression is limited to regions of the shoot and root meristems and developing vasculature (Fig 3G and 3H). *DEM1* mRNA was also observed in vegetative shoot meristems, developing anthers and ovules (Fig 3I, 3K and 3L), but was less detectable in inflorescence meristems (Fig 3J). *DEM2* mRNA was also detected in inflorescence meristems, developing anthers and ovules, but was more diffuse throughout these tissues than *DEM1* mRNA (Fig 3M and 3N).

### *DEM* genes are required for normal male meiosis and pollen viability

To characterize the biological function of the *DEM* genes in *Arabidopsis*, we analyzed the phenotype of independent *dem1* and *dem2* T-DNA insertion mutants in ecotypes Col-0 and Ws-0. Southern blot analysis confirmed the T-DNA insertions in *DEM1* and *DEM2* (S4A and S4B Fig), and northern blot analysis on floral bud tissue confirmed that *dem1* and *dem2* mutants in each ecotype lacked the respective mRNA transcript (S4C and S4D Fig). We used qRT-PCR on RNA extracted from floral buds to confirm that *dem1* and *dem2* mutants in the ecotype Col-0 genetic background lacked the corresponding mRNA transcript (Fig 2C). We also measured *DEM1* and *DEM2* expression in floral buds of the *dem2* and *dem1* mutants, respectively, to determine if mutation in one *DEM* gene could be compensated for by increased expression of the other *DEM* gene. There was a slight increase in the expression of the other *DEM* gene in both mutants, however, the increase was only statistically significant for *DEM2* expression in the *dem1* mutant (Fig 2C).

Plants homozygous for single *dem1* or *dem2* T-DNA insertion alleles in both Col-0 and Ws-0 ecotypes had no obvious phenotypic defects compared to the wild-type plants (S5A and S5B Fig). Subsequently, for both ecotypes, homozygous *dem1* and *dem2* plants were crossed to generate F1 plants heterozygous for both genes (*DEM1/dem1 DEM2/dem2*), and the F_2_ progeny and subsequent generations were characterized for phenotypic defects that correlated with the number of mutant *dem* alleles. Strikingly, no homozygous *dem1 dem2* double mutants were observed in the F_2_ or subsequent generations. Furthermore, *dem1/dem1 DEM2/dem2* plants were recovered in Col-0 but not in the Ws-0 genetic background. In total, we screened 153 F2 plants in the Ws-0 genetic background and failed to recover either homozygous *dem1 dem2* double mutant plants or *dem1/dem1 DEM2/dem2* plants.

*DEM1/dem1 DEM2/dem2* and *DEM1/dem1 dem2/dem2* plants in both genetic backgrounds, and *dem1/dem1 DEM2/dem2* plants in the Col-0 background showed no obvious phenotypic defects prior to reproductive development. However, segregation analysis of progeny produced by self-fertilization of these plants showed distortion in favour of the wild-type *DEM* alleles (S2 and S3 Tables), suggesting that the *dem1 dem2* genotype was transmitted through the male and/or female germline at a lower rate than other genotypes.

**Fig 4:**
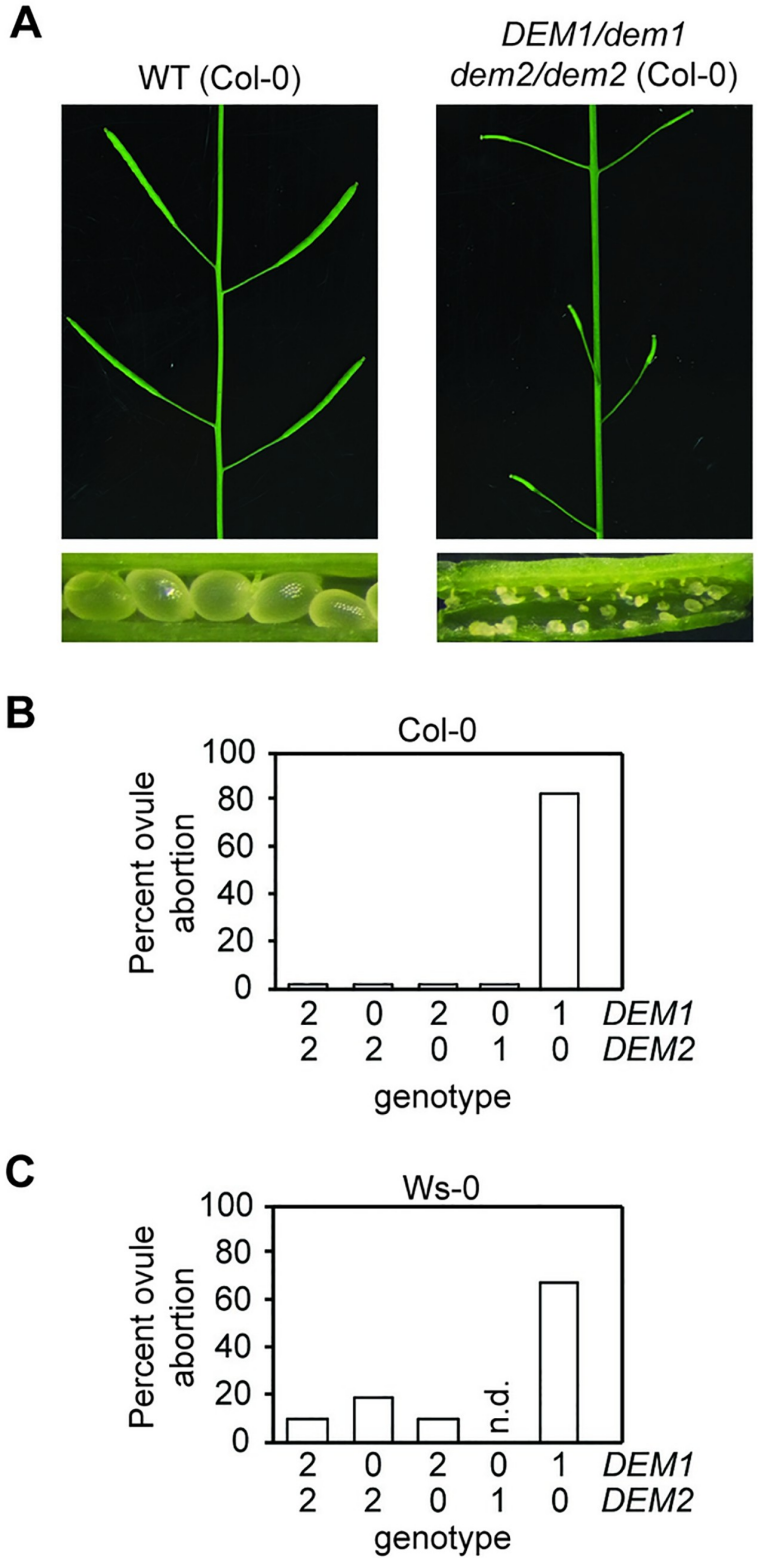
*DEM1* and *DEM2* are required for normal seed production in *Arabidopsis*. **(A-C)** Plants harboring only one wild-type *DEM1* allele (*DEM1/dem1 dem2/dem2*) in both the Col-0 and Ws-0 genetic backgrounds exhibited significant ovule abortion. **(A)** Comparison of wild-type (WT) and *DEM1/dem1 dem2/dem2* siliques of Col-0 genetic background. Note the occurrence of degenerated ovules in *DEM1/dem1 dem2/dem2* siliques compared to the appearance of normal seeds in WT siliques. **(B-C)** Percentage of aborted ovules in self-fertilized WT and *dem* genotypes for Col-0 **(B)** and Ws-0 **(C)** genetic backgrounds. At least 200 ovules were assayed, involving at least three separate plants for each genotype. X-axis of **(B)** and **(C)** represent the copy number of *DEM1* and *DEM2* wild-type alleles in each genotype. No *dem1/dem1 DEM2/dem2* plants were recovered in the Ws-0 genetic background; n.d., not determined.

**Fig 5:**
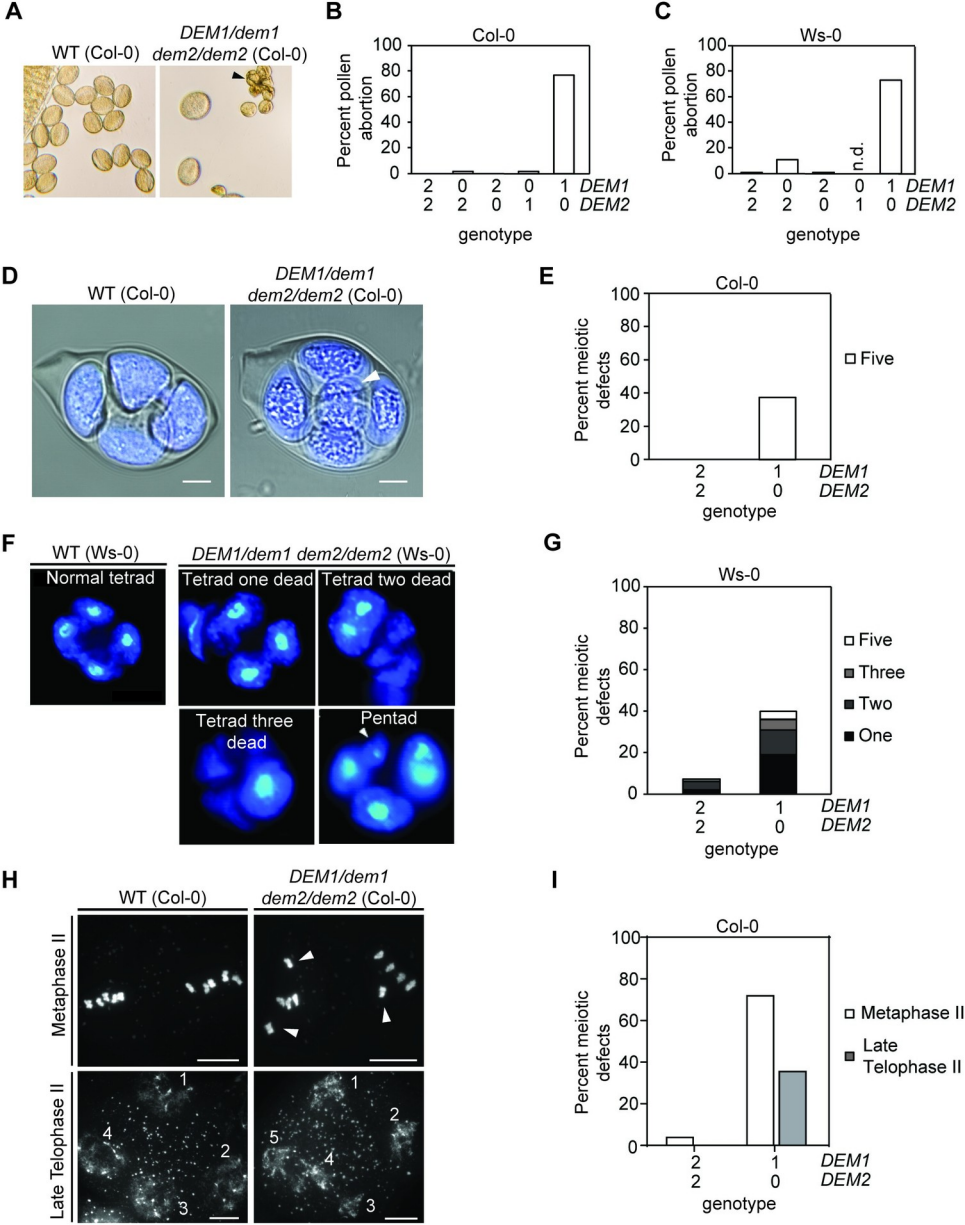
*DEM1* and *DEM2* are required for normal pollen development and male meiosis in *Arabidopsis*. **(A)** Phenotype of pollen produced by wild-type (WT) and *DEM1/dem1 dem2/dem2* plants in the Col-0 genetic background. Dead pollen grains are indicated by a black arrowhead. **(B-C)** Percentage of aborted mature pollen in WT and *dem* genotypes for the Col-0 **(B)** and Ws-0 **(C)** genetic backgrounds. At least 700 mature pollen in total were assayed, involving at least three separate plants for each genotype. n.d., not determined as *dem1/dem1 DEM2/dem2* were not recovered in the Ws-0 genetic background. **(D)** Confocal image of DAPI-stained tetrads, combined with a DIC imaging to view membrane structures, shows an excess of meiotic products (i.e. a pentad) in a *DEM1/dem1 dem2/dem2* plant (ecotype Col-0); an extra product of meiosis of diminished size is highlighted by the white arrowhead. Bar = 5 μm. **(E)** Percentage abnormal tetrads produced by WT and *DEM1/dem1 dem2/dem2* plants in the Col-0 background. At least 80 meiotic events were scored, involving at least three separate plants for each genotype. **(F)** DAPI-stained defective male tetrads of *DEM1/dem1 dem2/dem2* plants in ecotype Ws-0 showing one to three dead microspores, and an extra microspore of diminished size in a pentad (white arrowhead). **(G)** Percentage of defective tetrads in WT and *DEM1/dem1 dem2/dem2* plants of ecotype Ws-0. At least 200 tetrads were assayed for each genotype. **(H)** DAPI-stained meiotic chromosome spreads revealed misaligned chromosomes (white arrowheads) away from the metaphase plate at metaphase II in *DEM1/dem1 dem2/dem2* male meiocytes, and also shown is an example of five daughter cells produced at telophase II in *DEM1/dem1 dem2/dem2* male meiocytes (ecotype Col-0). **(I)** Percentage of male meiocytes showing defects at metaphase II (misaligned chromosomes) and late telophase II (pentads) in wild-type and *DEM1/dem1 dem2/dem2* plants in ecotype Col-0. At least 60 meiocytes were assayed, involving at least three separate plants for each genotype. X-axis of **(B), (C), (E), (G),** and **(I)** represent the copy number of *DEM1* and *DEM2* wild-type alleles in each genotype.

Among genotypes harboring multiple *dem* alleles, only *DEM1/dem1 dem2/dem2* plants exhibited major defects in fertility and had smaller siliques compared to the wild-type plants (Fig 4A). Dissection of mature siliques from the *DEM1/dem1 dem2/dem2* plants revealed ovule abortion rates of 60% and 80% in ecotypes Ws-0 and Col-0, respectively (Fig 4B and 4C), suggesting defects in pollen viability and/or ovule development. Indeed, the *DEM1/dem1 dem2/dem2* plants showed a high pollen abortion rate of 70–80% (Fig 5A–5C). While self-fertilization of *DEM1/dem1 dem2/dem2* plants produced a small amount of viable seed, these plants were completely ineffective in producing progeny when used as males in crosses to wild-type plants (Table 1), which was most likely due to a combination of the high pollen abortion rate, low viability of surviving pollen and ultimately, less viable pollen being attached to the stigma after manual crossing compared to self-pollination.

**Table 1:** Transmission of gametes depends on the dosage of functional *DEM* alleles in both the gametes and the plant that undergoes meiosis to produce the gametes. Segregation data from crosses involving *dem* mutations in the two genetic backgrounds (Col-0 and Ws-0) were analyzed separately and together for significance. The expected segregation of genotypes assuming equal transmission of wild-type *DEM* and mutant *dem* alleles is listed in brackets. *N*, number of progeny scored; *P*, probability for a chi-square distribution with three degrees of freedom and an expected segregation ratio of 1:1:1:1, or with one degree of freedom and an expected segregation ratio of 1:1. For some crosses, chi-square probabilities were also determined for observed and expected segregation ratios for just *dem1 DEM2* versus *DEM1 DEM2* gametes, and these probabilities are listed in the third row of the table for these crosses.

Cross (♀ x ♂)	*N*	Genotype of gametes transmitted				*P*
		*dem1* *dem2*	*DEM1* *dem2*	*dem1* *DEM2*	*DEM1* *DEM2*	
WT x *DEM1/dem1 dem2/dem2*^a^ (Col-0)	0^a^	n/a	n/a	n/a	n/a	n/a
WT x *DEM1/dem1 DEM2/dem2* (Col-0)	58	1 (14.5)	17 (14.5)	16 (14.5) (20)	24 (14.5) (20)	0.0002 0.21
WT x *DEM1/dem1 DEM2/dem2* (Ws-0)	39	2 (9.75)	12 (9.75)	6 (9.75) (12.5)	19 (9.75) (12.5)	0.0007 0.009
WT x *DEM1/dem1 DEM2/dem2*^b^ (Col-0 and Ws-0)	97	3 (24.25)	29 (24.25)	22 (24.25) (32.5)	43 (24.25) (32.5)	0.0001 0.009
WT x *DEM1/dem1 DEM2/DEM2* (Col-0)	52	n/a	n/a	29 (26)	23 (26)	0.41
*DEM1/dem1 dem2/dem2* x WT (Col-0)	63	24 (31.5)	39 (31.5)	n/a	n/a	0.06
*DEM1/dem1 DEM2/dem2* x WT (Col-0)	48	8 (12)	11 (12)	9 (12) (14.5)	20 (12) (14.5)	0.06 0.04
*DEM1/dem1 DEM2/dem2* x WT (Ws-0)	24	2 (6)	5 (6)	6 (6) (8.5)	11 (6) (8.5)	0.07 0.23
*DEM1/dem1 DEM2/dem2* x WT^b^ (Col-0 and Ws-0)	72	10 (18)	16 (18)	16 (18) (23)	30 (18) (23)	0.007 0.04
*DEM1/dem1 DEM2/DEM2* x WT (Col-0)	33	n/a	n/a	16 (16.5)	17 (16.5)	0.86

^a^ sterile as a male, no progeny was obtained from five separate experiments involving multiple *DEM1/dem1 dem2/dem2* plants

^b^combined data for Col-0 and Ws-0 backgrounds. n/a, not applicable.

It has been reported that up to 20% of T-DNA insertion mutants of *Arabidopsis* carry chromosomal translocations, and nearly all translocation lines when crossed to non-transgenic wild type show a pollen abortion rate of ~50% in heterozygous F1 plants [21]. To address the possibility that high pollen abortion rates in *DEM1/dem1 dem2/dem2* plants may due to chromosomal translocations in the T-DNA insertion mutants, we assessed pollen abortion rates in double heterozygous *DEM1/dem1 DEM2/dem2* F1 plants produced by crossing the single *dem1* and *dem2* mutants of Col-0. If chromosomal translocations were associated with either of the Col-0 T-DNA mutants, the pollen abortion rate would be expected to be at least 50% in the double heterozygous plants [21]. We assessed 717 pollen grains in total from three different *DEM1/dem1 DEM2/dem2* Col-0 plants and found a pollen abortion rate of ~6% compared to the pollen abortion rate of ~70% for *DEM1/dem1 dem2/dem2* Col-0 plants (Fig 5B). These data strongly indicate that the high pollen abortion rate observed in *DEM1/dem1 dem2/dem2* plants was due to the *dem* mutations rather than the possibility of chromosomal translocations in the T-DNA mutant lines.

The pollen abortion rate of ~70% in *DEM1/dem1 dem2/dem2* plants exceeded the expected number of *dem1 dem2* pollen (i.e. 50%; Fig 5A–5C) and indicated that a significant portion of *DEM1 dem2* pollen was also aborted. Furthermore, the low fertility of *DEM1/dem1 dem2/dem2* plants suggested that the surviving mature pollen also had substantially reduced viability, and that the absence of a wild-type *DEM2* allele in the *DEM1/dem1 dem2/dem2* sporophyte adversely affected the viability of *DEM1 dem2* pollen. Phenotypic analysis of additional *dem* genotypes revealed that pollen viability was not solely dependent on the pollen’s genotype but also the number of *DEM2* alleles in the parent plant that undergoes meiosis. Specifically, segregation distortion against *dem1 DEM2* pollen compared to *DEM1 DEM2* pollen was observed for *DEM1/dem1 DEM2/dem2* plants (*P* < 0.01) but not for *DEM1/dem1 DEM2/DEM2* plants (*P* = 0.41; Table 1). Thus, in addition to the pollen genotype, pollen viability is dependent on the number of functional *DEM2* alleles in the parent plant.

Confocal microscopy on DAPI-stained male tetrads from *DEM1/dem1 dem2/dem2* plants of ecotype Col-0 revealed that 38% of tetrads were irregular and had an excess of meiotic products (Fig 5D and 5E). In *DEM1/dem1 dem2/dem2* plants of ecotype Ws-0, tetrads with excessive meiotic products were observed, as well as degenerated tetrads with one or more dead microspores (Fig 5F and 5G).

To further investigate the role of *DEM* in male meiosis, we produced meiotic chromosome spreads of DAPI-stained *DEM1/dem1 dem2/dem2* male meiocytes for ecotype Col-0. Interestingly, 72% of the daughter cells produced by meiosis I in *DEM1/dem1 dem2/dem2* plants contained at least one chromosome that failed to align on the metaphase plate at metaphase II (Fig 5H and 5I). Furthermore, we confirmed that about a third of male meioses in *DEM1/dem1 dem2/dem2* plants of ecotype Col-0 produced five rather than four daughter cells at late telophase II (Fig 5H and 5I). All other phases of meiosis I and II in these *DEM1/dem1 dem2/dem2* plants were indistinguishable from wild type (S6 Fig).

As mentioned earlier, *dem1/dem1 DEM2/dem2* plants were recovered in Col-0 but not in Ws-0 background, and these Col-0 plants were indistinguishable from the wild-type plants in terms of pollen (Fig 5B) and ovule abortion rates (Fig 4B). Thus, in the Col-0 background at least, a single functional *DEM2* allele in *dem1/dem1 DEM2/dem2* plants was sufficient for normal vegetative development, fertility and seed production.

In summary, our phenotypic analysis of male reproductive development showed that *DEM* genes are required for male meiosis, pollen development and male gamete viability, but *DEM2* is more important than *DEM1*. This increased importance of *DEM2* correlated with significantly higher levels of *DEM2* mRNA compared to *DEM1* mRNA in pollen and sperm cells, and in other tissues of the plant (Fig 2).

### *DEM* genes are required for megaspore competitiveness and megagametophyte development

**Fig 6:**
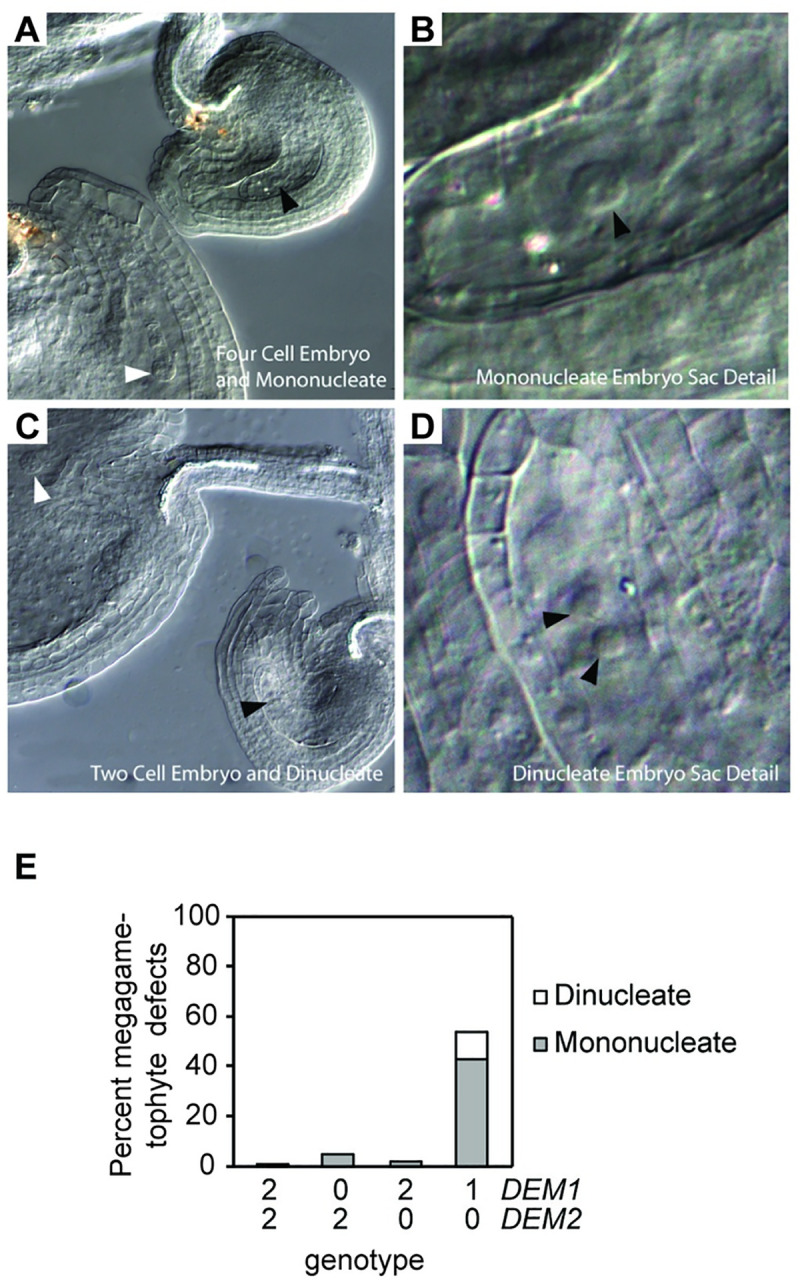
*DEM1* and *DEM2* are required for normal megagametophyte development in *Arabidopsis*. **(A-D)** Defective female gametophytes produced in *DEM1/dem1 dem2/dem2* plants (Ws-0 genetic background). Developing ovules were observed as cleared whole mounts. **(A-B)** Four cell embryo (white arrowhead) and mononucleate embryo sac (black arrowhead) in two adjacent ovules. **(C-D)** Two cell embryo (white arrowhead) and di-nucleate embryo sac (black arrowheads) in two adjacent ovules. **(E)** Percentage of mononucleate and di-nucleate embryo sacs in self-fertilized wild-type and *DEM1/dem1 dem2/dem2* plants of ecotype Ws-0. At least 200 ovules were assayed, involving at least three separate plants for each genotype. X-axis represents the copy number of *DEM1* and *DEM2* wild-type alleles in each genotype.

Crosses of *DEM1/dem2 DEM2/dem2* females to wild-type males revealed that the *dem1 dem2* genotype was transmitted through the female germline to the next generation two to five times less efficiently than *DEM1 DEM2*, depending on the Col-0 versus Ws-0 genetic background (Table 1). Furthermore, the *DEM1 dem2* and *dem1 DEM2* gametes were also transmitted through the female germline at a lower frequency than expected for equal segregation of wild-type and mutant alleles (Table 1). Combining the data for Col-0 and Ws-0 together, the results demonstrated that the segregation distortion against *dem* mutations through the female germline was highly significant (*P* < 0.01; Table 1). However, despite the reduced co-transmission of *dem1* and *dem2* alleles, a significant portion of *dem1 dem2* genotypes were still transmitted to the next generation (Table 1). Furthermore, at a stage when ovules of wild-type plants were fertilized and contained developing embryos, self-fertilized *DEM1/dem1 dem2/dem2* Ws-0 plants had approximately 50% of embryo sacs arrested before the first or second round of post-meiotic mitosis (Fig 6). Thus, high ovule abortion rate observed in *DEM1/dem1 dem2/dem2* plants (Fig 4) is most likely due to defects in both pollen (Fig 5A–5C) and embryo sac viability (Fig 6).

As was the case for pollen, there was a significant segregation distortion against *dem1 DEM2* female gametes compared to *DEM1 DEM2* female gametes for *DEM1/dem1 DEM2/dem2* plants (*P* < 0.05) but not for *DEM1/dem1 DEM2/DEM2* plants (*P* = 0.86; Table 1). Therefore, megagametophyte viability is also dependent on the number of functional wild-type *DEM2* alleles in both the megagametophyte and in the parent plant that undergoes meiosis to produce the megagametophyte.

### Transgenic complementation of gamete viability defects

Despite the PCR genotyping of many hundreds of plants, we never recovered homozygous *dem1 dem2* double mutants, most likely due to substantially reduced viability of *dem1 dem2* gametes and/or a potentially lethal phenotype of homozygous *dem1 dem2* double mutant embryos. As mentioned earlier, *dem1/dem1 DEM2/dem2* Col-0 plants showed almost no pollen abortion (Fig 5B), however when self-fertilized, the progeny showed strong segregation distortion in favour of the wild-type *DEM2* allele (S3 Table; P < 0.05). To demonstrate transgenic complementation of the gamete viability defects associated with *dem* mutants, we introduced a wild-type *DEM1* transgene into the *dem1/dem1 DEM2/dem2* mutant of Col-0. Initially, we transformed the homozygous *dem1* mutant with binary vector pUQC10043 carrying the *DEM1* transgene linked to the selectable marker gene *BAR* (S10 Fig), which confers resistance to the herbicide Basta. A T1 plant carrying the *DEM1* transgene was selected and crossed to the homozygous *dem2* mutant. In the F2 generation from this cross, we identified a *dem1/dem1 DEM2/dem2* plant carrying the *DEM1* transgene, and then screened the F3 progeny of this plant for Basta resistant *dem1 dem2* double mutants. Five of the 23 Basta-resistant F3 progeny that we genotyped with PCR tests were homozygous for both the *dem1* and *dem2* mutations. The segregation ratio of 5:18 for homozygous *dem1 dem2* mutants to other genotypes (*dem1/dem1 DEM2/dem2* or *dem1/dem1 DEM2/DEM2*) fits a 1:3 segregation ratio expected for Mendelian inheritance (P = 0.72). Thus, the *DEM1* transgene complemented the gamete viability defects in the *dem1/dem1 DEM2/dem2* mutant and allowed the recovery of *dem1 dem2* double mutants.

The *dem1 dem2* double mutant plants carrying the *DEM1* transgene were confirmed by Southern blot analysis (S4B Fig), and these plants showed a very similar phenotype to *DEM1/dem1 dem2/dem2* plants (Figs 4, 5B and 5C), i.e., normal vegetative development (S5A Fig) and a high pollen and ovule abortion rate (S5B and S5C Fig). These results suggest that the *DEM1* transgene could compensate for the absence of *DEM1* but not the absence of *DEM2* in the *dem1 dem2* double mutant.

### DEM and RAS-RELATED NUCLEAR PROTEIN 1 (RAN1) interact *in vitro* and co-localize during male meiosis and pollen development

**Fig 7:**
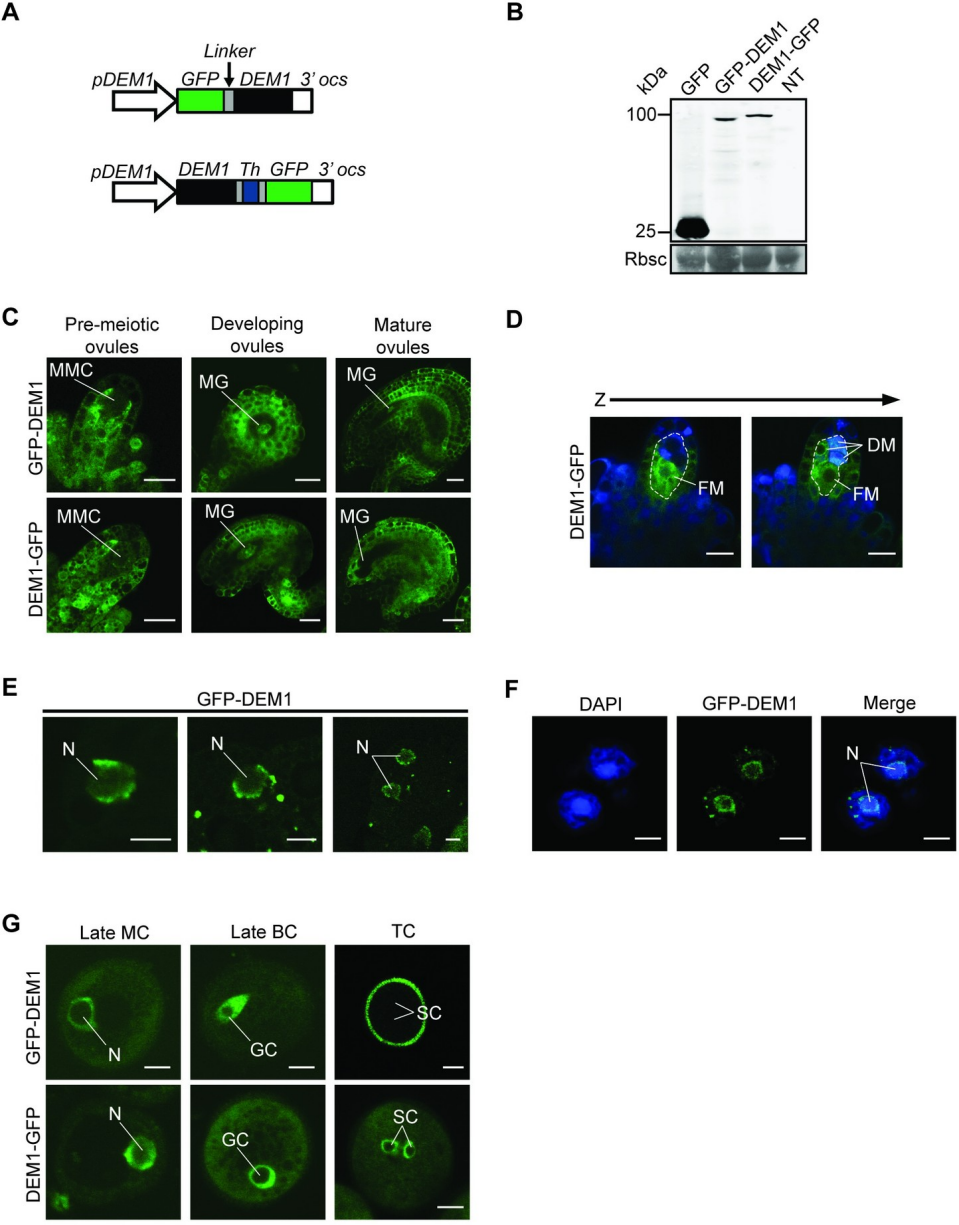
GFP-tagged DEM1 is expressed in developing gametophytes and sporophytic tissues of *Arabidopsis*. **(A)** Transgenes used to express DEM1 N-terminal or C-terminal GFP fusion proteins under the control of the *DEM1* promoter (*pDEM1*). The coding sequence for six alanine residues (*Linker*) were inserted between *GFP* and *DEM1* of *pDEM1*:*GFP-DEM1*. Similarly, the *pDEM1*:*DEM1-GFP* transgene contained the coding sequence for three alanine residues, followed by a thrombin cleavage site and three more alanine residues (*Th*). **(B)** Anti-GFP western blot on floral bud extracts of *35S*:*GFP* (GFP), *pDEM1*:*GFP-DEM1* (GFP-DEM1) and *pDEM1*:*DEM1-GFP* (DEM1-GFP) transgenic lines, and a non-transgenic control (NT). Molecular weight is indicated in kDa. The molecular masses of GFP-DEM1 and DEM1-GFP are approximately 100 kDa, and of GFP is 27 kDa. The Coomassie Brilliant Blue-stained (CBB) membrane shows Rubisco (Rbsc) as a loading control. **(C)** Ubiquitous and punctate GFP-tagged DEM1 expression in ovules prior to meiosis (pre-meiotic ovules), and in developing and mature post-meiotic ovules. In pre-meiotic ovules, very little GFP fluorescence could be seen in the megaspore mother cell (MMC), whereas high expression was observed in the sporophytic cells surrounding the MMC (bar = 10 μm). In developing ovules, high GFP expression is observed in the sporophytic tissue and in the megagametophyte (MG) (bar = 20 μm). In mature ovules, high GFP fluorescence was observed in the sporophytic tissues, but very little expression was seen in the megagametophyte (MG) (bar = 20 μm). **(D)** Two optical sections (Z-stack) showing high expression of DEM1-GFP in the functional megaspore (FM) of an ovule (bar = 10 μm). DM, dead megaspores. **(E)** Expression of GFP-DEM1 in male pre-meiotic cells; GFP-DEM1 localized around the nuclear envelope (bar = 5 μm). N, nucleus. **(F)** Expression of GFP-tagged DEM1 was also detected in meiotic products of male meiosis; two cells of a tetrad are shown in the figure. Note the subcellular localization of GFP-DEM1 around the nuclear envelope relative to the DAPI stained nucleus (N) (bar = 5 μm). **(G)** Expression of GFP-tagged DEM1 during pollen development (bar = 5 μm). N, nucleus of monocellular pollen; GC, generative cell; SC, sperm cells; MC, monocellular pollen; BC, bicellular pollen; TC, tricellular pollen.

To investigate the intracellular localization of DEM proteins, transgenic lines of *Arabidopsis* were produced that expressed *pDEM1*:*GFP-DEM1* or *pDEM1*:*DEM1-GFP* (Fig 7A). These transgenic lines expressed the full-length fusion proteins of ~100 kDa in size (Fig 7B). Both *pDEM1*:*GFP-DEM1* and *pDEM1*:*DEM1-GFP* were expressed in all developing tissues examined, including developing pollen and ovules (Fig 7C–7G) and differentiating root tips (S7 Fig). Furthermore, the fusion proteins localized to cytoplasm and around the nuclear envelope, where they overlapped with DAPI staining of nuclear DNA (Figs 7D, 7F and S7).

**Fig 8:**
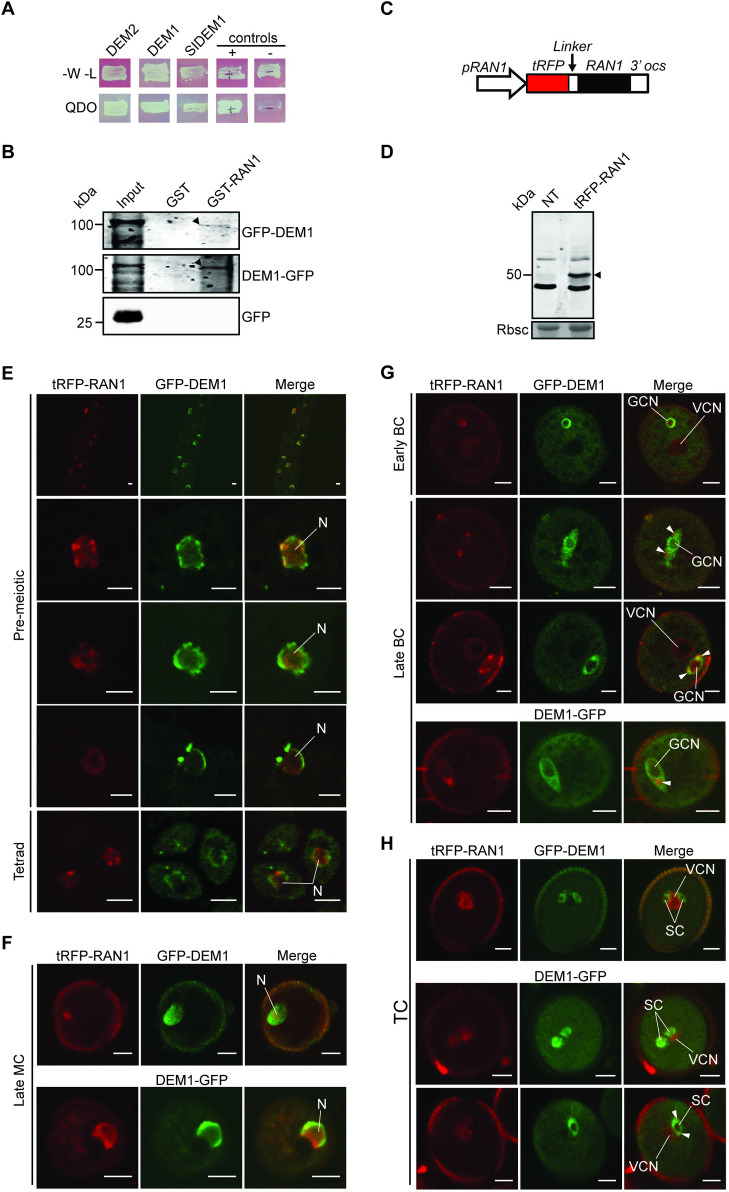
DEM interacts with RAN1, and the proteins co-localize during male meiosis and pollen development. **(A)** RAN1 interacts with *Arabidopsis* DEM1 and DEM2, and tomato DEM1 (SlDEM1) in the yeast two-hybrid system. Yeast were plated on SD media lacking tryptophan and leucine (-W-L) to select for the bait (DEM) and prey (RAN1) vectors and show equal viability of all strains. Yeast were also plated on quadruple dropout media (QDO) to test for interactions between the bait and the prey. Known interacting (+) and non-interacting (-) controls are also shown. **(B)** GST-RAN1, but not GST, precipitates 100 kDa GFP-DEM1 and DEM1-GFP from floral bud extracts (black arrowheads). GFP-expressing plants were included to rule out binding of RAN1 to GFP (lower panel). Input represents an anti-GFP western of floral bud extracts from each transgenic line. **(C)** Transgene used to express RAN1 N-terminal tagRFP (tRFP) fusion protein under the control of the *RAN1* promoter (*pRAN1*). The coding sequence for six glycine residues (*Linker*) were inserted between *tRFP* and *RAN1*. **(D)** Anti-tRFP western blot of *pRAN1*:*tRFP-RAN1* transgenic and non-transgenic (NT) floral bud extracts. The tRFP-RAN1 is approximately 50 kDa (black arrowhead). The CBB membrane shows Rubisco (Rbsc) as a loading control. **(E-H)** Expression and co-localization of GFP-tagged DEM1 and tagRFP-RAN1 (tRFP-RAN1) during male meiosis and pollen development in wild-type ecotype Col-0 plants. **(E)** GFP-DEM1 localized around the nuclear envelope with additional weak signal throughout the nucleus and cytoplasm of pre-meiotic cells and tetrads, whereas tRFP-RAN1 typically localized to the nucleus (N). We examined 18 pre-meiotic cells in total, all 18 cells expressed detectable tagRFP-RAN1 and GFP-DEM1 and showed a similar localization of the fusion proteins. Representative pre-meiotic cells are shown in the figure. **(F)** Monocellular microspores (MC) show localization of GFP-tagged DEM1 around the nuclear envelope and tRFP-RAN1 to the nucleus, including the nuclear periphery. **(G)** Bicellular pollen showing co-localization of tRFP-RAN1 and GFP-tagged DEM1 around the nuclear envelope and in extranuclear foci at opposite poles of the generative cell (white arrowheads). **(H)** In tricellular pollen, GFP-tagged DEM1 was expressed in sperm cells (SC) around the nuclear envelope and throughout the sperm cytoplasm, whereas RFP-tagged RAN1 was primarily expressed in the immediately adjacent vegetative nucleus. In some tricellular pollen, weak RAN1 fluorescence overlapped with strong DEM1 fluorescence in extranuclear foci at opposite poles of sperm cells (lower panel; white arrowheads). We examined 93 pollen in total and 74 (80%) expressed detectable tagRFP-RAN1, 45 (48%) expressed detectable GFP-DEM1 or DEM1-GFP, and 39 (42%) expressed both tagRFP-RAN1 and GFP-DEM1 or DEM1-GFP (**E-H**). Pollen at the same stage expressing both tagRFP-RAN1 and GFP-DEM1 or DEM1-GFP showed a similar localization of the fusion proteins, and representative pollen are shown in the figure (**E-H**). Bar = 5 μm. BC, bicellular pollen; TC, tricellular pollen; GCN, generative cell nucleus; VCN, vegetative cell nucleus.

To gain insight into DEM’s molecular function, a yeast two-hybrid system was employed to screen *Arabidopsis* cDNA libraries for proteins that interact with *Arabidopsis* DEM2. Six strongly interacting prey vectors were identified (S4 Table), and upon sequencing, were all shown to carry *Arabidopsis RAS-RELATED NUCLEAR PROTEIN 1* (*RAN1*) cDNA sequences (S4 Table). Each of these six *RAN1* clones represented one or the other of two unique overlapping clones encoding the C-terminal 92 and 186 amino acids of the predicted 221-amino acid RAN1 protein (S4 Table). The longest RAN1 prey interacted with both *Arabidopsis* DEMs and full-length tomato DEM1 as bait in the yeast two-hybrid system (Fig 8A).

To further investigate a potential interaction between DEM1 and RAN1, GST-RAN1 was expressed and purified from *E*. *coli*, and then used as bait in pull-down experiments with floral buds extracts from transgenic lines of *Arabidopsis* expressing *pDEM1*:*GFP-DEM1* or *pDEM1*:*DEM1-GFP* (Fig 8B). The GST-RAN1 pull down assay successfully precipitated the 100 kDa GFP-fusion proteins that corresponded to the molecular weight of full-length GFP-DEM1 and DEM1-GFP (Fig 8B). Furthermore, GST alone did not precipitate GFP-DEM1 or DEM1-GFP, and GST-RAN1 did not precipitate GFP (Fig 8B).

A transgenic line of wild-type *Arabidopsis* ecotype Col-0 was produced that expressed a *pRAN1*:*tRFP-RAN1* transgene and a full-length ~50 kDa tRFP-RAN1 fusion protein in floral buds (Fig 8C and 8D). The tRFP-RAN1 fusion protein was also detected during microsporogenesis and pollen development, primarily in the nucleus of cells, but also in perinuclear and extranuclear foci in microspores and the generative cell of bicellular pollen, respectively (S8 Fig). To determine if DEM1 and RAN1 had the potential to interact during male meiosis and in developing pollen, the transgene *pRAN1*:*tRFP-RAN1* was co-expressed along with *pDEM1*:*GFP-DEM1* or *pDEM1*:*DEM1-GFP* in wild-type ecotype Col-0 (Fig 8E–8H). GFP-tagged DEM1 and tRFP-RAN1 co-localized around the nuclear envelope in pre-meiotic cells, tetrads (Fig 8E) and monocellular microspores (Fig 8F). In early bicellular pollen, GFP-tagged DEM1 and tRFP-RAN1 co-localized around the nuclear envelope of the generative cell, and in late bicellular pollen, co-localized to the aforementioned extranuclear foci at opposite poles of the generative cell (Fig 8G). In tricellular pollen, GFP-tagged DEM1 was expressed in sperm cells around the nuclear envelope and throughout the cytoplasm, whereas RFP-tagged RAN1 was primarily expressed in the immediately adjacent vegetative nucleus (Fig 8H). In some tricellular pollen, weak RAN1 fluorescence also overlapped with strong DEM1 fluorescence in extranuclear foci at opposite poles of sperm cells (Fig 8H, lower panel). No background fluorescence was observed in non-transgenic pre-meiotic cells or gametophytes except for in the pollen coat (S9 Fig).

Unlike the *pDEM1*:*DEM1* transgene (S4 and S5 Figs), *pDEM1*:*DEM1-GFP* and *pDEM1*:*GFP-DEM1* transgenes did not allow the recovery of *dem1/dem1 dem2/dem2* double mutant plants (S5 Table). However, the *DEM1-GFP* and *GFP-DEM1* transgenes suppressed the extent of pollen and ovule abortion in the *DEM1/dem1 dem2/dem2* genotype (S5D Fig), and alleviated the distorted segregation ratio against *DEM1/dem1 dem2/dem2* plants compared to *DEM1/DEM1 dem2/dem2* plants in progeny of self-fertilized *DEM1/dem1 dem2/dem2* plants (S5 Table). Crosses between *DEM1/dem1 dem2/dem2* plants carrying the *GFP-tagged DEM1* transgenes as males to wild-type female plants produced a limited number of progeny, indicating that the transgenes increased pollen fertility in *DEM1/dem1 dem2/dem2* plants (S6 Table). Not all of the progeny produced from these crosses inherited the transgene, suggesting that the *GFP-tagged DEM1* transgenes enhanced gamete viability in *DEM1/dem1 dem2/dem2* plants by functioning in both pollen and the transgenic parent plant that underwent meiosis to produce the pollen (S6 Table).

## Discussion

Prior to our current study, the *DEM1* gene of tomato was the only member of the *DEM* gene family to be genetically characterized in plants [10]. Tomato *DEM1* is expressed in all differentiating tissue, and the *dem1* mutant of tomato has dysfunctional meristems resulting in a seedling lethal phenotype [10]. However, it was unclear whether *DEM1* played a fundamental role in cell division or alternatively, had a more specific role in defining the identity of meristematic cells in tomato. In our current work, we used *Arabidopsis* as a more tractable genetic model to investigate a possible fundamental role of *DEM* genes in cell division in plants. In contrast to the *dem1* mutant of tomato, both the *dem1* and *dem2* single mutants of *Arabidopsis* were indistinguishable from wild type in both vegetative development and reproductive fertility, but a fundamental role for the *DEM* genes in cell division, gamete viability and fertility was revealed in *DEM1/dem1 dem2/dem2* plants carrying mutations in both *DEM1* and *DEM2*. In contrast to *DEM1/dem1 dem2/dem2* plants, *dem1/dem1 DEM2/dem2* plants showed normal levels of fertility, indicating that *DEM2* plays a more important role than *DEM1* in gamete viability. The increased importance of *DEM2* gamete viability correlated with higher mRNA levels of *DEM2* compared to *DEM1* in most tissues examined and particularly in the vegetative shoot apex, developing siliques, pollen and sperm (Fig 2).

### *DEM* gene expression in *Arabidopsis* is consistent with a function in cell division

*DEM1* and/or *DEM2* transcripts are enriched in tissues undergoing cell division, including the shoot apex, flowers and developing siliques (Fig 2A). The *in situ* expression pattern of *DEM* genes in differentiating tissues appeared punctate, particularly for *DEM1* (Fig 3), which is typical of cell cycle-regulated genes such as histones, cyclins and cyclin-dependent kinases [22–25]. In support of these findings, *DEM1* has been shown previously to be up-regulated in cell cultures during the mitosis phase of the cell cycle [26] and in actively dividing shoot apices [27]. Expression of DEM1 fused to GFP also appeared punctate in differentiating tissues (Figs 7C and S7). Interestingly, GFP-DEM1 and DEM1-GFP were observed in newly formed sperm cells but not in the vegetative cell of pollen, which does not undergo further cell division (Fig 8H). Together, these findings suggest that *DEM1* expression is up-regulated immediately prior to, during and just after cell division.

### *DEM1/dem1 dem2/dem2* mutants have defects in male meiosis, pollen and embryo sac development

*DEM1/dem1 dem2/dem2* plants produced defective tetrads with an excess of irregular-sized meiotic products after male meiosis (Fig 5D–5G). Upon performing meiotic chromosome spreads on *DEM1/dem1 dem2/dem2* male meiocytes, we found that chromosomes were often misaligned on the metaphase plate at metaphase II (Fig 5H and 5I). Despite the misalignment of chromosomes in ~70% of daughter cells of *DEM1/dem1 dem2/dem2* male meiocytes at metaphase II, chromosome segregation during anaphase II and early telophase II appeared normal (S6 Fig). However, we cannot rule out the possibility of anaphase II defects in *DEM1/dem1 dem2/dem2* plants because aberrant three-dimensional organisation of chromosomes at anaphase II would be difficult to discern in the chromosome spreads. While the misaligned chromosomes at metaphase II moved towards the poles during meiosis II, they were often not partitioned properly into four distinct daughter nuclei at late telophase II, thereby resulting in the formation of pentads (Fig 5D–5G), and in the case of ecotype Ws-0, also degenerated tetrads (Fig 5F and 5G). In addition to defects in male meiosis and pollen development, we also observed approximately 50% of embryo sacs arrested before the first or second round of post-meiotic mitosis in *DEM1/dem1 dem2/dem2* plants (Fig 6).

In contrast to *DEM1/dem1 dem2/dem2* plants, *dem1/dem1 DEM2/dem2* plants recovered in the Col-0 genetic background had very few aborted pollen (Figs 4B and 5B). These results suggest an increased importance of *DEM2* compared to *DEM1* particularly in pollen development, which correlated with significantly higher expression of *DEM2* compared to *DEM1* in pollen and sperm (Fig 2B).

While *dem1/dem1 DEM2/DEM2* plants had wild-type phenotypes in both ecotypes, *dem1/dem1 DEM2/dem2* plants were not recovered in the Ws-0 background. These results could indicate that a single functional *DEM2* allele in *dem1/dem1 DEM2/dem2* plants in the Ws-0 background is insufficient for embryo viability, or alternatively, that the *dem2* T-DNA insertion allele may interfere with expression of the wild-type *DEM2* allele in heterozygous Ws-0 embryos.

### Sporophytic and gametophytic expression of *DEM* contributes to gamete viability

*DEM* expression during microgametogenesis is essential for pollen viability, as the transmission of the *dem1 dem2* genotype through the male germline was almost completely abolished in *DEM1/dem1 dem2/dem2* and *DEM1/dem1 DEM2/dem2* plants (Table 1). However, *DEM1/dem1 DEM2/dem2* plants with one functional copy of *DEM2* also transmitted a lower than expected proportion of *dem1 DEM2* gametes compared to plants with two functional copies of *DEM2* (i.e. *DEM1/dem1 DEM2/DEM2*; Table 1), suggesting the expression of *DEM2* in the diploid parent plant that undergoes meiosis contributes to pollen development. In support of these findings, we showed that *DEM1/dem1 dem2/dem2* plants produce a much higher rate of pollen abortion (~70–80%) than expected (50%) if only *dem1 dem2* pollen were aborted (Fig 5A–5C). It is therefore likely that in the absence of *DEM2*, a significant portion of *DEM1 dem2* pollen are also aborted in *DEM1/dem1 dem2/dem2* plants due to insufficient expression of *DEM1* in the diploid microspore mother cell that undergoes meiosis or in other supporting sporophytic tissue around the developing pollen. Consistent with the importance of *DEM1* gene expression in the sporophyte in the absence of *DEM2*, we found that *GFP-DEM1* and *DEM1-GFP* transgenes partially restored pollen fertility in *DEM1/dem1 dem2/dem2* plants even when the pollen did not inherit the transgene (S6 Table). One possible explanation for these sporophytic effects is that *DEM* transcripts or DEM protein produced by the microspore mother cell or other sporophytic cells are passed into the products of meiosis and function during gamete formation.

In the Col-0 genetic background, *dem1/dem1 DEM2/dem2* plants were indistinguishable from wild type, whereas *DEM1/dem1 dem2/dem2* plants had a wild-type phenotype during vegetative development but showed defects in pollen and embryo sac development (Figs 5, 6 and S5). It is interesting to note that *DEM2* mRNA but not the *DEM1* mRNA has been found in the phloem [28] and is graft-transmissible [29]. Graft-transmissible mRNAs are often highly expressed [29], and *DEM2* mRNAs levels were significantly higher than *DEM1* mRNA levels in all tissues and cells examined, except for microdissected ovules and unpollinated pistils (Fig 2). Future research should investigate whether the *DEM2* transcript has the capacity to move cell-to-cell during reproductive development.

There was also evidence for both sporophytic and gametophytic expression of *DEM* genes contributing to embryo sac viability (Table 1 and Fig 6), but the impact of *dem* mutations on female reproductive development was less severe than on pollen development. *DEM1/dem1 DEM2/dem2* plants transmitted the *dem1 dem2* genotype through the megagametophyte at a reduced rate, indicating that *dem1 dem2* megagametophytes were viable but less likely to become the surviving megaspore following meiosis. The improved viability of *dem1 dem2* embryo sacs compared to *dem1 dem2* pollen could be due to a larger amount of sporophyte-derived cytoplasm being transferred to the products of female meiosis (i.e. embryo sacs) compared to the products of male meiosis (i.e. pollen).

### Potential interaction between DEM and RAN around the nuclear envelope and in extracellular foci

Our results indicate that DEM’s role in cell division during meiosis, and in pollen and embryo sac development, could be mediated through an interaction with RAN1. RAN proteins are members of the RAS superfamily of small GTPase proteins and is highly conserved within eukaryotes [30–32]. In *Arabidopsis*, three *RAN* genes (*RAN1*, *RAN2* and *RAN3*) have been identified with very high sequence identity between themselves and their orthologues in animals and yeasts [31,33,34]. RAN functions as a molecular switch, converting between a guanosine triphosphate (GTP)-bound form (RAN-GTP) and a guanosine diphosphate (GDP)-bound form (RAN-GDP) [35]. The location of these two nucleotide-bound forms of RAN within the cell is crucial to the function of the protein. In animal nuclei, high concentrations of RAN-GTP are generated due to the action of a chromatin-anchored RAN guanine nucleotide exchange factor (RanGEF), which removes GDP from RAN and replaces it with GTP [35,36]. In plants, a functional equivalent of RanGEF is yet to be identified [37,38]. RAN’s intrinsic GTPase activity is very low, but it is strongly activated by RanGTPase activating protein (RanGAP) [39–40]. During interphase in all eukaryotic cells, RanGAP is anchored to the cytoplasmic face of the nuclear envelope and ensures that any RAN-GTP leaving the nucleus is immediately hydrolyzed to RAN-GDP in the cytoplasm [40–43]. The GTPase function of RAN is also influenced by its interaction with RAN binding proteins (RanBPs) at the nuclear envelope [44–48]. Together, the subcellular positioning of these effectors with RAN generates a steep RAN-GTP gradient relative to chromatin and across the nuclear envelope [39,42,48]. This spatial distribution of the RAN-GTP gradient drives many additional processes in eukaryotic cells, including spindle assembly during cell division, reformation of the nuclear envelope following cell division and nucleocytoplasmic transport [32,49–57].

The cell division defects in *dem* meiocytes, pollen (Fig 5) and embryo sacs (Fig 6), along with disorganized patterns of cell division in meristems of tomato *dem1* mutants [10], are consistent with disruption of RAN-associated processes. DEM1 and RAN1 co-localize around the nuclear envelope of pre-meiotic cells and in pollen (Fig 8), and future research should investigate a potential role of DEM proteins in RAN-dependent breakdown of the nuclear envelope, formation of the spindle apparatus during cell division and/or reformation of the nuclear envelope following cell division. Besides co-localizing around the nuclear envelope, DEM1 and RAN1 co-localized transiently in extranuclear foci at opposite poles of the generative and sperm cells of developing pollen, but the nature and function of these foci also requires further investigation. While transient co-localization occurred during male meiosis and pollen development, DEM1 and RAN1 fusion proteins were also observed at unique locations within cells (Fig 8). These differences in subcellular localization of DEM1 and RAN1 suggest that, in addition to their interaction, these proteins are likely to have unique and independent cellular functions.

DEM1 fusion proteins showed an uneven distribution throughout the cytoplasm of differentiating cells in a pattern resembling the endoplasmic reticulum (ER) (Figs 7, 8 and S7) [58]. The nuclear envelope is a double membrane that forms a continuous membrane system with the ER, but in both plants and animals, the nuclear envelope undergoes a cycle of disassembly and reformation during cell division whereas the ER network remains intact [59–60]. Furthermore, Anderson and Hetzer [61] have demonstrated in the metazoan *Xenopus* that nuclear envelope formation and expansion are driven by chromatin-mediated shaping of the ER network after cell division.

Our transgenic work focusing on *DEM1* was initiated prior to our results showing that *DEM2* plays a more important role than *DEM1* in male gamete viability. However, future work should concentrate on the further characterization of *DEM2*’s role in meiosis and gamete viability, and the importance of DEM2’s interaction with RAN1 in these processes. Additionally, it will be important to investigate whether the apparent increased importance of *DEM2* compared to *DEM1* in gamete viability is due to *DEM2*’s higher expression in sporophytic and gametophytic tissues and cells, or alternatively, due to functional differences between the DEM1 and DEM2 proteins. Given that the *DEM* genes are expressed in tissues undergoing high rates of cell division, it would also be interesting to assess *dem* mutants for mitotic phenotypes and changes in mitotic indices in meristems, and for DNA content of somatic cells.

In conclusion, we have shown that *DEM* genes play a key role in cell division and gamete viability in *Arabidopsis*, which most likely involves an interaction with RAN1. The presence of DEM homologues in diverse eukaryotic lineages suggests a conserved and critical cell division function of *DEM*-like genes in many eukaryotes. Alternatively, the region of conservation may relate to an interaction motif or domain of DEM-like proteins, and the biological roles of the proteins in diverse eukaryotic lineages could be distinct. The region of most similarity across eukaryotes corresponds to amino acids 253–458 of *Arabidopsis* DEM1, and this aligns with amino acids 40–226 of *S*. *pombe* DEM-like protein (S3 Fig), which when fused to GFP localizes to the nuclear rim [14]. These conserved amino acids residues may have a function in localizing DEM-like proteins to the nuclear rim, thus allowing DEM-like proteins to conduct biological functions that may be crucial to cell division or other cellular processes in many eukaryotic species.

## Materials and methods

### Phylogenetic analysis of DEM-like proteins

To identify proteins with similarity to DEM1, BLAST searches [62] using the tomato DEM1 sequence (NP_001234563.1) queried against the NCBI database were undertaken. Protein sequences from sequenced and annotated genomes from various eukaryota lineages were identified for including in multiple sequence alignments. Multiple sequence alignments of protein sequences were undertaken using Multiple Sequence Comparison by Log-Expectation (MUSCLE) from EMBL-EBI with default parameters. Rooted phylogenetic trees were constructed using the Maximum Likelihood method with Bootstrap analysis (1000 replication) and Poisson model in MEGA6 software [63].

### Plant handling, growth conditions and transformation

#### *Arabidopsis thaliana* genotypes

Two independent T-DNA insertion mutants for both *DEM1* (AT4G33400) and *DEM2* (AT3G19240) were used in this study. T-DNA insertion mutants *dem1-2* (SALK_016893C) and *dem2-2* (SALK_070099) in the Columbia (Col-0) ecotype were obtained from the Arabidopsis Biological Resource Center (ABRC, The Ohio State University, Columbus, OH). T-DNA insertion mutants *dem1-1* and *dem2-1* in the Wassilewskija (Ws-0) ecotype were obtained from the Wisconsin Biotechnology Center [64,65]. Techniques to confirm the presence or absence of T-DNA insertions in *DEM1* and *DEM2* are described in S1 Text. Details on production of transgenic *Arabidopsis* Col-0 lines expressing GFP and tagRFP fusion proteins are provided in S1 Text. The oligonucleotides used to genotype *Arabidopsis* T-DNA insertion mutants are listed in S7 Table.

#### *Arabidopsis* growth conditions

Seeds of *Arabidopsis* were germinated on damp University of California (UC) soil mix or on MS plates. Seeds were then stratified at 4°C for four to five days before growing at 21°C under 16-hour photoperiods of fluorescent lighting (70–80 μmol/m^2^/s).

#### *Arabidopsis* crossing and progeny analysis

For crossing of *Arabidopsis*, sepals, petals and anthers were removed from suitable flowers of the female parent. Anthers were obtained from newly opened flowers from the male plant and pollen was transferred to the pistil on the carpel of the female parent by direct physical contact. Emasculation controls that were not pollinated were included in crossing experiments as controls. Specific plant genotypes were identified by PCR genotyping.

#### *Arabidopsis* seed collection

Seeds were collected after the plants were fully desiccated and turned brown. Siliques were gently crushed and the released seeds were sifted through a tea strainer to remove silique debris. Seeds were stored in 1.5 ml Eppendorf tubes with a small hole in the lid (made with a 0.45 mm needle) and placed in containers with silica gel desiccant to keep the seeds dry and prevent contamination by fungi or bacteria.

#### *Arabidopsis* transformation

The *Agrobacterium* T-DNA binary vectors used in this study are shown in S10 Fig. A single colony of *Agrobacterium tumefaciens* strain GV3101 containing the appropriate T-DNA binary vector was inoculated into 5 ml LB (Luria-Bertani) medium (1% tryptone, 0.5% yeast extract, 1% NaCl; w/v) with 50 μg/ml gentamicin, 50 μg/ml kanamycin and 50 μg/ml rifampicin and pre-cultured for 48 hours at 28°C. About 1 ml of pre-culture was then inoculated into 200 ml LB containing the same antibiotics. The 200 ml culture was incubated for 24 hours at 28°C and centrifuged at 5000 rpm at 4°C for 15 minutes in a Beckman JA-10 rotor.

The floral dip method [66] was used to transform *Arabidopsis*. The *Agrobacterium* pellet was resuspended in 500 ml of 5% (w/v) sucrose containing 187.5 μl of Silwet L-77 (Lehle Seeds, USA). Stems with open floral buds of *Arabidopsis* were dipped and mildly agitated in the *Agrobacterium* solution for five seconds. The plants were then covered for 24 hours to maintain high humidity.

#### Selection of transgenic *Arabidopsis* plants

Depending on the plant selectable marker, T_1_ plants were selected in soil for Basta resistance or MS plates for kanamycin resistance. For selecting T_1_ plants in soil, T_1_ seeds collected from the transformed plants were evenly spread over a damp UC soil mix and stratified for four to five days at 4°C. After germination, the seedlings were sprayed with Basta herbicide (0.04% w/v) at two-day intervals. To select for kanamycin resistance, sterile T_1_ seeds were plated on MS plates containing 75 μg/ml kanamycin, stratified for four to five days at 4°C and then incubated vertically in the growth room.

### *In situ* hybridization

*In situ* hybridization experiments were performed on *Arabidopsis* wild-type ecotype Ws-0, as described by Jackson [67] and Vielle-Calzada *et al*. [68]. Antisense DIG-labelled RNA was produced to full-length *DEM1* (AT4G33400) and *DEM2* (AT3G19240) cDNAs, which were ordered from the *Arabidopsis* Biological Resource Center (clones G2D10T7 and ATTS5957, respectively).

### RNA extraction, gel blot analysis and quantitative real-time reverse transcriptase PCR (qRT-PCR)

For Northern gel blots, total RNA was extracted from *Arabidopsis* closed floral buds younger than stage 13 [69] as previously described [70–71]. Bundles of fresh or frozen *Arabidopsis* closed floral buds were collected and crushed with a mortar and pestle under liquid nitrogen into fine powder without any pre-treatment. Total RNA was further purified to remove contaminants using 1-butanol [70]. Northern gel blots were conducted as described previously [71]. For qRT-PCR analysis, *Arabidopsis* plants (ecotype Columbia) were grown to the four-leaf stage (shoot apex tissue, young and mature leaves) or to the mature plant stage at 2 weeks after flowering (cauline stems, flowers, floral buds and developing siliques) at 24°C day/20°C night and photoperiods of 8 hours (four-leaf stage plants) or 16 hours (mature plants) light (170 μE m^-2^ s^-1^). At least three independent samples from each tissue were collected from between three and 100 plants to provide replicate RNA samples for statistical analysis of qRT-PCR assays. Tissue was ground in liquid nitrogen and total RNA was prepared using the SV Total RNA Isolation System (Promega, Madison WI, USA) or TRIzol according to the manufacturer’s instructions (Invitrogen, Life Technologies, Carlsbad, CA, USA). cDNA was produced from 1 μg of total RNA and subsequently used as template (using the equivalent of 20 ng RNA per reaction) for real-time RT-PCR experiments as described previously [72]. The primer pair 5’-ATAACTAGTGGTCCGGTCCATCAC-3’ / 5’-TGGTCATTGCAACGCCTATG-3’ was used for detection of *DEM1* transcript levels in various wild-type tissues, except for floral buds (S7 Table). To avoid detection of truncated *DEM1* transcripts in the *dem1* mutant, the primer pair 5’-CCCCGTCTACTCAGAACTCAGC-3’ / 5’-CGACTTTCAAAATCCACCAT-3’ was used for measuring *DEM1* mRNA levels in floral buds of wild-type, *dem1* and *dem2* plants (S7 Table). The primer pair 5’-CCTTCTCCGGTAACAAATCGC-3’ / 5’-AAAGTCGTTTTCCAGAGAGGCTG-3’ was used for detection *DEM2* transcript levels in all genotypes and tissues tested (S7 Table). Expression detected from three *β-actin* genes of *Arabidopsis* [72] was used an internal standard to normalise the qRT-PCR data (S7 Table).

### DNA extraction and gel blot analysis

High purity genomic DNA was purified and used in Southern gel blot analysis [73] to confirm the presence or absence of T-DNA insertions in *DEM1* and *DEM2*. Radioactive probes were generated as for RNA gel blot analysis.

### Gametophyte analysis

Microspores were analyzed by dissecting anthers and staining in 1 μg/ml DAPI (4’, 6-diamino-2-phenylindole), as described elsewhere [74]. Mature pollen was dissected from anthers immediately prior to anthesis and microscopy was used to score the pollen for viability based on appearance (e.g. Fig 5A). Female gametophytes and embryos were analyzed by clearing with Hoyer’s solution [75]. These were then assayed under a ZeissAxioskop 2 compound microscope equipped with a ZeissAxiocam digital camera using florescence, bright field and differential interference contrast (DIC) optics.

### Cytology

Meiotic chromosome spreads were performed as described by Ross *et al*. [76].

### Yeast two-hybrid screen

The Matchmaker Library Construction and Screening kit (Clontech Laboratories Inc., California) was used to construct *Arabidopsis* ecotype Col-0 cDNA libraries and screen them with bait vectors containing the *DEM1* and *DEM2* cDNAs. Further details are provided in S1 Text.

### Expression and purification of GST-RAN1 and protein binding assays

The *Arabidopsis RAN1* coding region (AT5G20010) was expressed as a GST-tagged fusion protein in *E*. *coli*. Experimental procedures used to express GST-tagged RAN1 and pull-down DEM1-GFP and GFP-DEM1 from transgenic plant extracts are provided in S1 Text.

### SDS-PAGE and immunoblotting

Protein samples for immunoblotting were snap-frozen in liquid nitrogen, ground into a fine powder with pestle, briefly thawed with the addition of loading buffer and then treated at ~95°C for 5 minutes. Protein samples were run on 10% SDS-PAGE gels and transferred from the SDS-PAGE to a PVDF membrane (Bio-Rad) following the manufacturer’s protocol. For immunoblotting, anti-His mouse (Amersham Biosciences) or anti-GFP mouse (Roche Applied Science) IgG_1_ monoclonal primary antibodies were diluted 1000-fold and the secondary Alexa fluor 680 anti-mouse IgG (Invitrogen) were diluted 5000-fold. Blocking reagents were purchased from LI-COR Biosciences and blocking, binding and washing steps were as described in LI-COR protocol document number 988–09288. For visualization of membranes, the Odyssey infra-red scanner was used. Total protein was visualized by staining gels and membranes with Coomassie Brilliant Blue (R250).

### Confocal scanning laser microscopy (CSLM) and image analysis

A Zeiss LSM 510 Meta (Carl Zeiss, Germany) confocal laser-scanning microscope (CSLM) was used with a 40 x, 1.3 oil-immersion plan neofluar objective for root tips, ovules, pollen and tetrads. GFP was visualized by excitation with an argon laser at 488 nm and detection with a 505–530 nm band-path filter. RFP was visualized using a HeNe1 laser at 543 nm and detection with a 560 nm long-path filter and DAPI was visualized using a diode laser for 405 nm and detection with a 420–480 nm band-pass filter. The multi-track scan mode was used for co-localization images. Samples for confocal microscopy were dissected and placed in PBS buffer with 10% (v/v) glycerol and kept on ice prior to viewing. For staining nuclei, 2.5 μg/ml DAPI was used and samples kept on ice for 1–5 minutes prior to viewing.

### Statistical analysis

All statistical tests were implemented in RStudio using custom R scripts. Multiple corrections of generated *p*-values were carried out using the Benjamini & Hochberg method.

## Supporting information

S1 Fig Phylogenetic tree (rooted with the *Giardia* sequences) of plant DEM and DEM-like proteins found in diverse eukaryote lineages. Software and parameters used in the analysis are described in Materials and Methods. The name of the organism is indicated on the tree, followed by the accession number. Scale bar indicates 0.05 substitutions per site. The plant DEM proteins represented in this tree (Viridiplantae) are the same as those used in Fig 1.
(TIF)

S2 Fig Multiple sequence alignment of plant and non-plant DEM-like proteins. Multiple alignments on full-length DEM and DEM-like protein sequences were undertaken using the Multiple Sequence Comparison by Log-Expectation (MUSCLE) tool from EMBL-EBI and visualized using the Multiple Sequence Alignment Viewer from NCBI. Plant DEM homologues are highly conserved with increased level of identity in the C-terminal half of the proteins. Grey boxes represent amino acids in agreement with the consensus of the multiple sequence alignment.
(TIF)

S3 Fig *Arabidopsis* DEM1 (NP_195066.1) contains an amino acid domain with high similarity to a nuclear rim localization domain of VID27-like protein from S*chizosaccharomyces pombe* (BAA87237.1). Pairwise sequence alignment of NP_195066.1 and BAA87237.1 was conducted using the LALIGN tool from EMBL-EBI.
(TIF)

S4 Fig DNA and RNA gel blot analysis of *dem1* and *dem2* T-DNA insertion mutants, and complemented *dem1 dem2* double mutant. **(A)** Gene structure of *DEM1* and *DEM2*, along with T-DNA insertions (open triangles), *HindIII* restriction sites (H3), and location of probes (grey boxes) used for DNA and RNA gel blot analysis. *dem1-1* and *dem2-1* are T-DNA insertion alleles in Ws-0 ecotype, while *dem1-2* and *dem2-2* are T-DNA insertion alleles in ecotype Col-0. **(B)** DNA gel blots on replicate DNA extractions confirmed T-DNA insertions in *dem1* and *dem2* mutants in Ws-0 and Col-0 genetic backgrounds, and in a *dem1 dem2* double mutant (Col-0) complemented with a *DEM1* transgene driven by its own promoter *pDEM1* (*dem1+2 comp*). DNA blots for Ws-0 lines were hybridized to the *DEM1 3’* probe (upper panel LHS), whereas the blots for the Col-0 lines were hybridized to the *DEM1 5’* probe (upper panels RHS). DNA gel blots were all hybridized to the *DEM2 5’* probe (lower panel). The T-DNA insertion alleles for the Col-0 *dem1* and *dem2* mutants are indicated by arrowheads. **(C)** RNA gel blot analysis of floral buds using the *DEM1* and *DEM2 5’* probes confirmed T-DNA knock-out of both endogenous *dem* genes, and over-expression of the *pDEM1*:*DEM1* transgene in the complemented Col-0 *dem1 dem2* double mutant (*dem1+2 comp*). Lower panel shows the 25S rRNA loading control. The two lanes for the complemented Col-0 *dem1 dem2* double mutant in **(B)** and **(C)** represent two separate plants of a transgenic line that was phenotypically normal during vegetative development and partially fertile. **(D)** RNA gel blot analysis of floral buds using the *DEM1 3’* probe confirmed the absence of full-length transcripts in Ws-0 *dem1* plants.
(TIF)

S5 Fig Defective phenotypes of *dem* mutants were partially complemented by *DEM1*, *GFP-DEM1* and *DEM1-GFP* transgenes. **(A)** Image showing 6-week old plants of wild type (WT) plants, *dem1* and *dem2* single mutants, *dem1/dem1 DEM2/dem2*, *DEM1/dem1 dem2/dem2*, and *dem1 dem2* double mutant complemented with *pDEM1*:*DEM1* transgene (*dem1+2 comp*) in Col-0 genetic background. **(B)** Normal seed production was observed in *dem1* and *dem2* single mutants and in *dem1/dem1 DEM2/dem2* plants, but not in *dem1/dem1 dem2/dem2* double mutant plants complemented with a *pDEM1*:*DEM1* transgene (*dem1+2 comp*). **(C)** Percent ovule, pollen and tetrad defects in *DEM1/dem1 dem2/dem2* plants (*dem*) and *dem1/dem1 dem2/dem2* double mutant complemented with a *pDEM1*:*DEM1* transgene (*dem1+2 comp*). At least 200 ovules, 500 pollen grains and 70 tetrads were assayed for each genotype. **(D)** Partial complementation of ovule and pollen abortion rates in *DEM1/dem1 dem2/dem2* (*dem*) plants hemizygous for the *pDEM1*:*GFP-DEM1* (GFP-DEM1) or *pDEM1*:*DEM1-GFP* (DEM1-GFP) transgene. Non-transgenic *DEM1/dem1 dem2/dem2* (*dem*) and wild-type (WT) plants were included as controls. At least 200 ovules and 700 pollen grains were assessed for each genotype.
(TIF)

S6 Fig DAPI-stained meiotic chromosome spreads of defective *DEM1/dem1 dem2/dem2* (Col-0) male meiocytes. **(A)** Pachytene. **(B)** Diakinesis. **(C)** Metaphase I. **(D)** Anaphase I. **(E)** Telophase I. **(F)** Anaphase II. **(G)** Early telophase II. Bar = 10μm.
(TIF)

S7 Fig Confocal laser scanning microscopy (CLSM) image of a root tip cells expressing GFP:DEM1 and DEM1:GFP fusion proteins under the control of the *DEM1* promoter. **(A)** Root tips of transgenic seedlings expressing GFP-DEM1 (left) or DEM1-GFP (right); bar = 20 μm. **(B)** DAPI-stained root tip cells expressing GFP-DEM1 or DEM1-GFP showing cytoplasmic and nuclear envelope localization; bar = 10 μm. **(C)** Localisation of GFP-DEM1 and DAPI in daughter cells of a root tip epidermal cell that has just divided. **(D-E)** Root tip expressing GFP:DEM1. **(E)** Higher magnification of sub-cellular localization of GFP:DEM1 in root tips corresponding to inset in **(D),** showing expression of GFP:DEM1 relative to the nucleus. Bar represents 50 μm for panels **(A-D)**, and 10 μm for panels **(E).** Left-hand side, centre and right-hand side panels correspond to DAPI-stained, GFP and merged DAPI/GFP images, respectively (**B-E**). Images are representative of at least five independent transgenic lines for each transgene.
(TIF)

S8 Fig Expression and subcellular localization of tRFP-RAN1 during male meiosis and male gametogenesis in transgenic *Arabidopsis*. DAPI staining and localization of tRFP-RAN1 at various stages of microgametophyte development. In tetrads and free monocellular microspores (MC), tRFP-RAN1 predominantly co-localized with DAPI in the nucleus (N), but was concentrated in peripheral nuclear foci, particularly in early MC microgametophytes (white arrowhead). In early to mid-stage bicellular (BC) pollen, tRFP-RAN1 was mainly located in the generative cell nucleus (GCN) and weak expression was detected in the vegetative cell nucleus (VCN). In late BC pollen, tRFP-RAN1 was concentrated in extranuclear foci adjacent to the generative cell nucleus (white arrowhead; GCN), and a weak signal was detected in the vegetative cell nucleus. In tricellular (TC) pollen, tRFP-RAN1 was predominantly located in the vegetative cell nucleus (VCN). Bar = 5 μm.
(TIF)

S9 Fig Lack of auto-fluorescence in most cell types in reproductive tissues of non-transgenic plants. **(A)** GFP auto-fluorescence was not detected in pre-meiotic and mature ovules. **(B)** GFP auto-fluorescence was detected in the pollen coat, but not inside pollen grains. **(C)** GFP auto-fluorescence was not detected in pre-meiotic cells or tetrads. **(D)** RFP and GFP auto-fluorescence was not detected in the DAPI stained nuclei of pre-meiotic cells, monocellular and bicellular pollen. Auto-fluorescence of RFP and particularly GFP was detected in the pollen coat of monocellular and bicellular pollen. Bar = 5 μm.
(TIF)

S10 Fig T-DNA regions of binary vectors used in this study. T-DNA binary vectors described in this study are all derived from the binary vectors pUQC477 and pUQC214 [71]. *35S* represents the Cauliflower Mosaic Virus *35S* promoter. The vector pUQC10332 is a derivative of pUQC214 with the *35S*:*GFP* transgene removed, and it has a single *NotI* site upstream of the *BAR* selectable marker for inserting additional transgenes. pUQC10847 was constructed by removing the *35S*:*BAR* sequence from pUQC477 [71], leaving a *NPTII* gene driven by the *Agrobacterium NOPALINE SYNTHASE* promoter (*NOS*) as a kanamycin selectable marker linked to a *NotI* site (for cloning in additional transgenes). *OCS 3’*, *OCTOPINE SYNTHASE 3’* terminator.
(TIF)

S1 Table Prediction of N-myristoylation (MYR) sites in DEM-like sequences in plants. (DOCX)

S2 Table Segregation analysis of wild-type *DEM* and mutant *dem* alleles in F_2_ progeny derived from crossing homozygous *dem1* and homozygous *dem2* single mutants. (DOCX)

S3 Table Segregation analysis of wild-type *DEM* and mutant *dem* alleles in progeny produced by self-fertilization of plants with one functional allele of *DEM*. (DOCX)

S4 Table RAN1 interacts with *Arabidopsis* DEM2 in the yeast two-hybrid system. (DOCX)

S5 Table Partial complementation of distorted segregation ratios by the *GFP-DEM1* and *DEM1-GFP* transgenes in *Arabidopsis* ecotype Col-0. (DOCX)

S6 Table Partial complementation of defective *dem* pollen by GFP-tagged *DEM1* transgenes. (DOCX)

S7 Table Oligonucleotides used in this study. (DOCX)

S1 Text Supplementary materials and methods. (DOCX)
