# Supplementary figures and images for: DEFECTIVE EMBRYO AND MERISTEMS genes are required for cell division and gamete viability in Arabidopsis
Source: PLoS Genet. 2021 May 17;17(5):e1009561. doi: 10.1371/journal.pgen.1009561 (PMC8158957; doi:10.1371/journal.pgen.1009561)

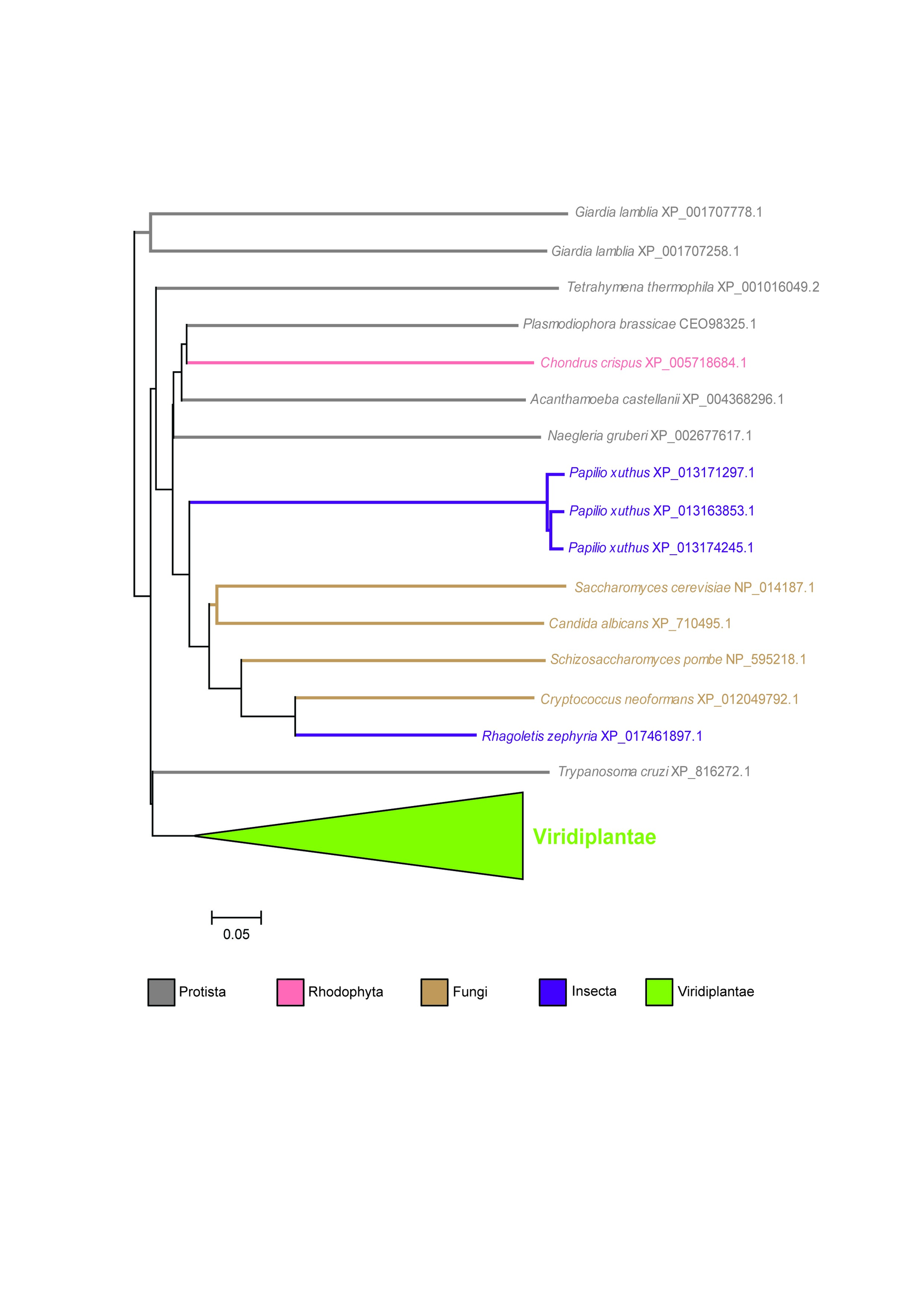

Supplement: S1 Fig — Software and parameters used in the analysis are described in Materials and Methods. The name of the organism is indicated on the tree, followed by the accession number. Scale bar indicates 0.05 substitutions per site. The plant DEM proteins represented in this tree (Viridiplantae) are the same as those used in Fig 1. (TIF) [file pgen.1009561.s001.tif]

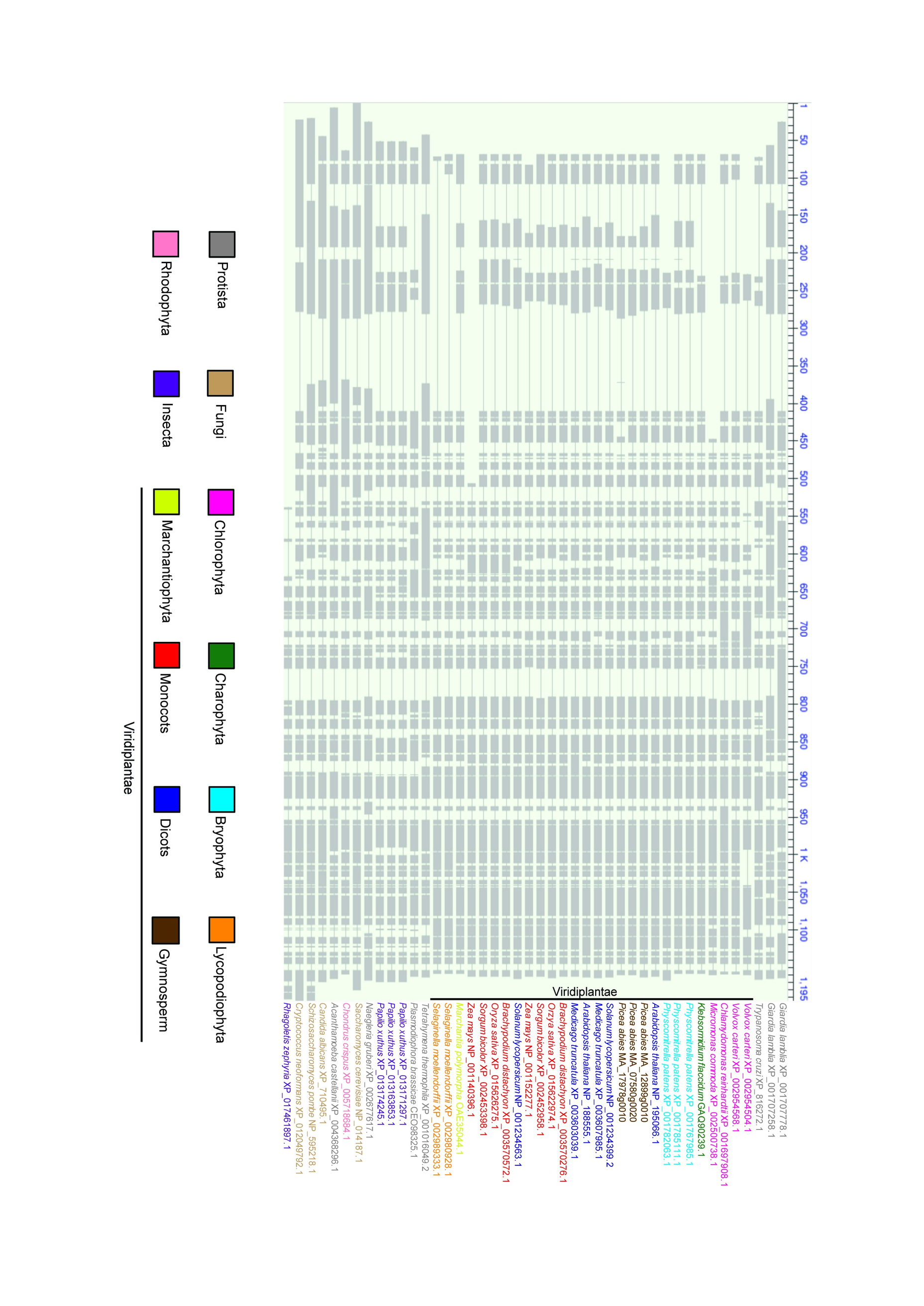

Supplement: S2 Fig — Multiple alignments on full-length DEM and DEM-like protein sequences were undertaken using the Multiple Sequence Comparison by Log-Expectation (MUSCLE) tool from EMBL-EBI and visualized using the Multiple Sequence Alignment Viewer from NCBI. Plant DEM homologues are highly conserved with increased level of identity in the C-terminal half of the proteins. Grey boxes represent amino acids in agreement with the consensus of the multiple sequence alignment. (TIF) [file pgen.1009561.s002.tif]

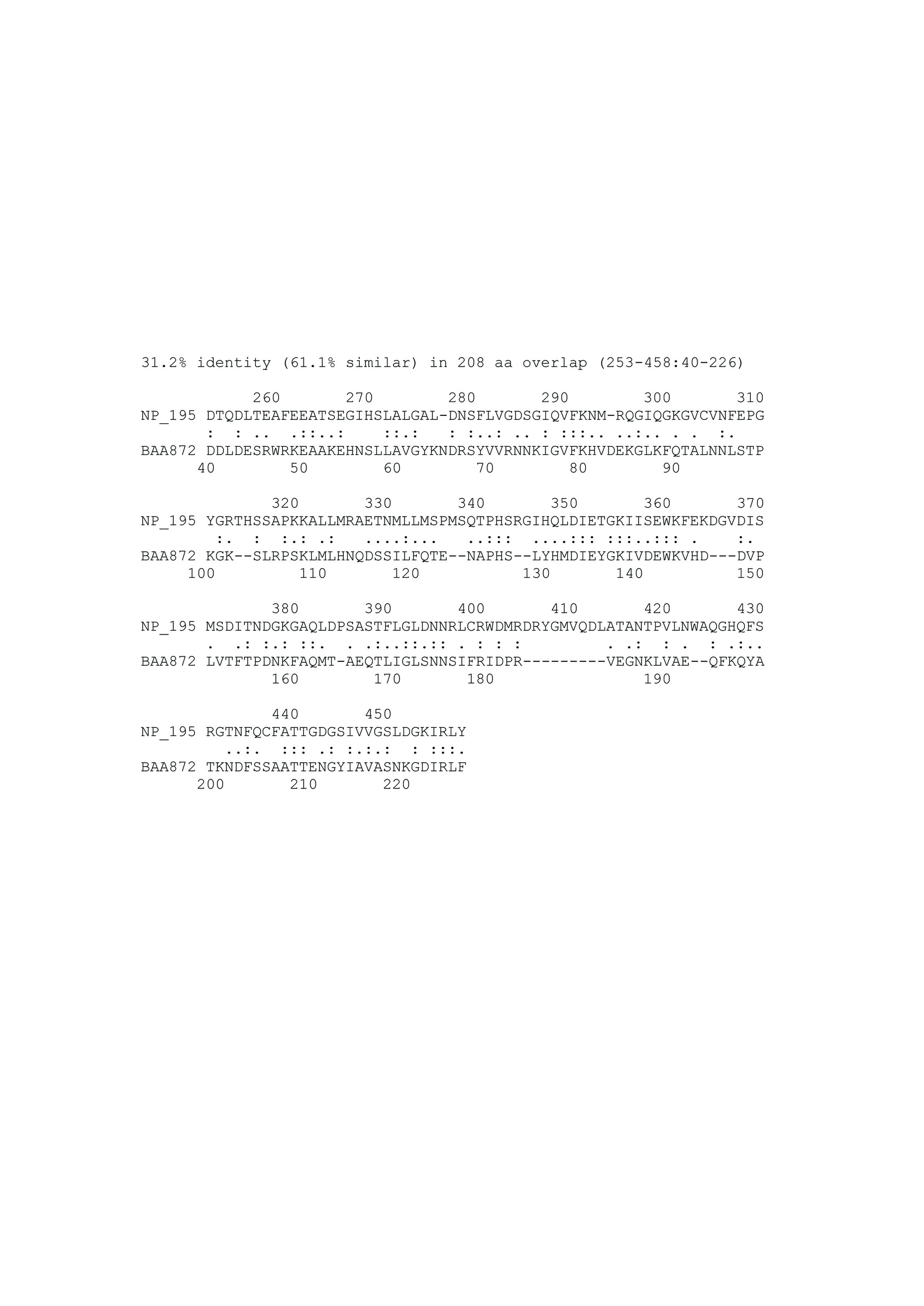

Supplement: S3 Fig — Pairwise sequence alignment of NP_195066.1 and BAA87237.1 was conducted using the LALIGN tool from EMBL-EBI. (TIF) [file pgen.1009561.s003.tif]

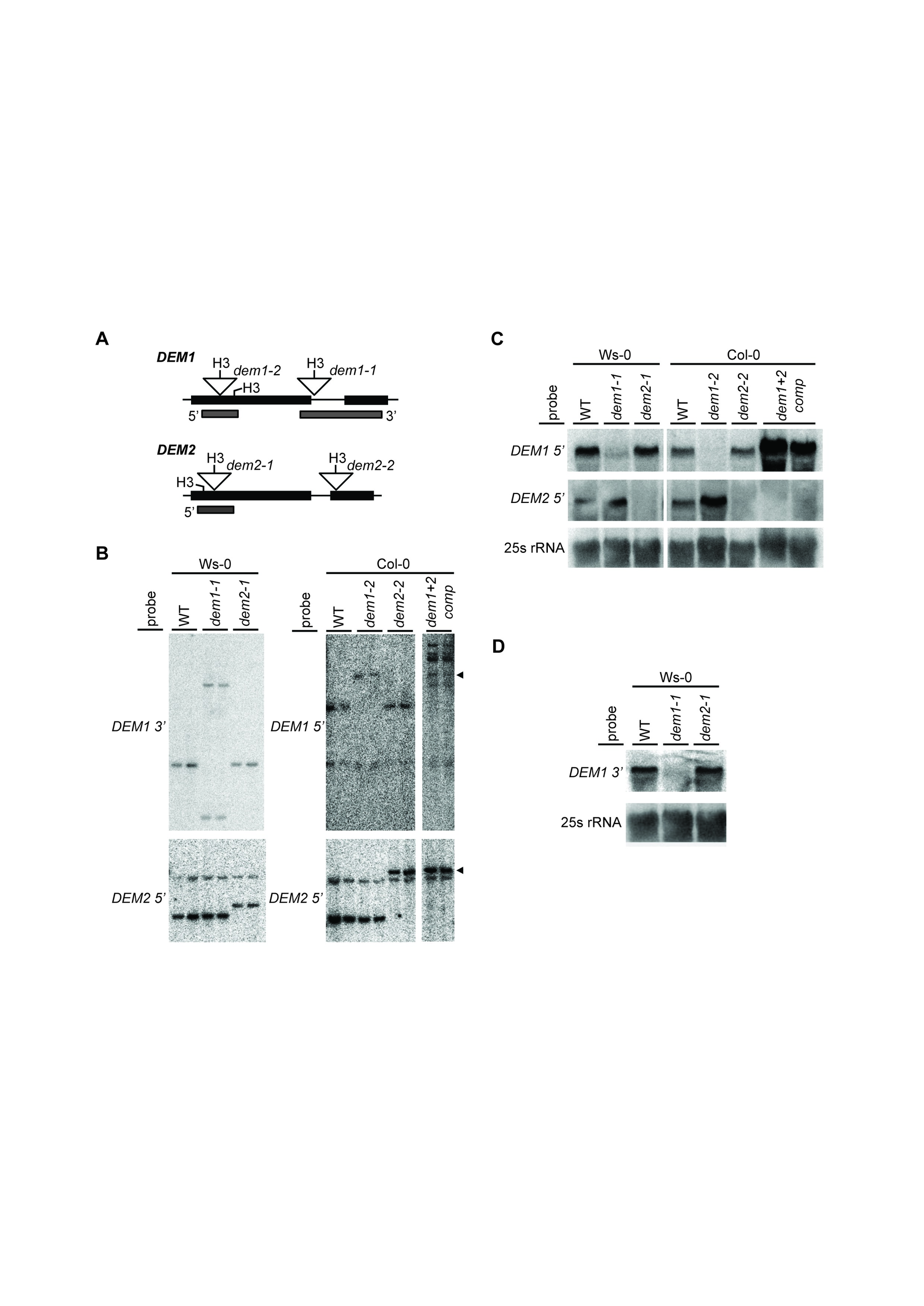

Supplement: S4 Fig — (A) Gene structure of DEM1 and DEM2, along with T-DNA insertions (open triangles), HindIII restriction sites (H3), and location of probes (grey boxes) used for DNA and RNA gel blot analysis. dem1-1 and dem2-1 are T-DNA insertion alleles in Ws-0 ecotype, while dem1-2 and dem2-2 are T-DNA insertion alleles in ecotype Col-0. (B) DNA gel blots on replicate DNA extractions confirmed T-DNA insertions in dem1 and dem2 mutants in Ws-0 and Col-0 genetic backgrounds, and in a dem1 dem2 double mutant (Col-0) complemented with a DEM1 transgene driven by its own promoter pDEM1 (dem1+2 comp). DNA blots for Ws-0 lines were hybridized to the DEM1 3’ probe (upper panel LHS), whereas the blots for the Col-0 lines were hybridized to the DEM1 5’ probe (upper panels RHS). DNA gel blots were all hybridized to the DEM2 5’ probe (lower panel). The T-DNA insertion alleles for the Col-0 dem1 and dem2 mutants are indicated by arrowheads. (C) RNA gel blot analysis of floral buds using the DEM1 and DEM2 5’ probes confirmed T-DNA knock-out of both endogenous dem genes, and over-expression of the pDEM1:DEM1 transgene in the complemented Col-0 dem1 dem2 double mutant (dem1+2 comp). Lower panel shows the 25S rRNA loading control. The two lanes for the complemented Col-0 dem1 dem2 double mutant in (B) and (C) represent two separate plants of a transgenic line that was phenotypically normal during vegetative development and partially fertile. (D) RNA gel blot analysis of floral buds using the DEM1 3’ probe confirmed the absence of full-length transcripts in Ws-0 dem1 plants. (TIF) [file pgen.1009561.s004.tif]

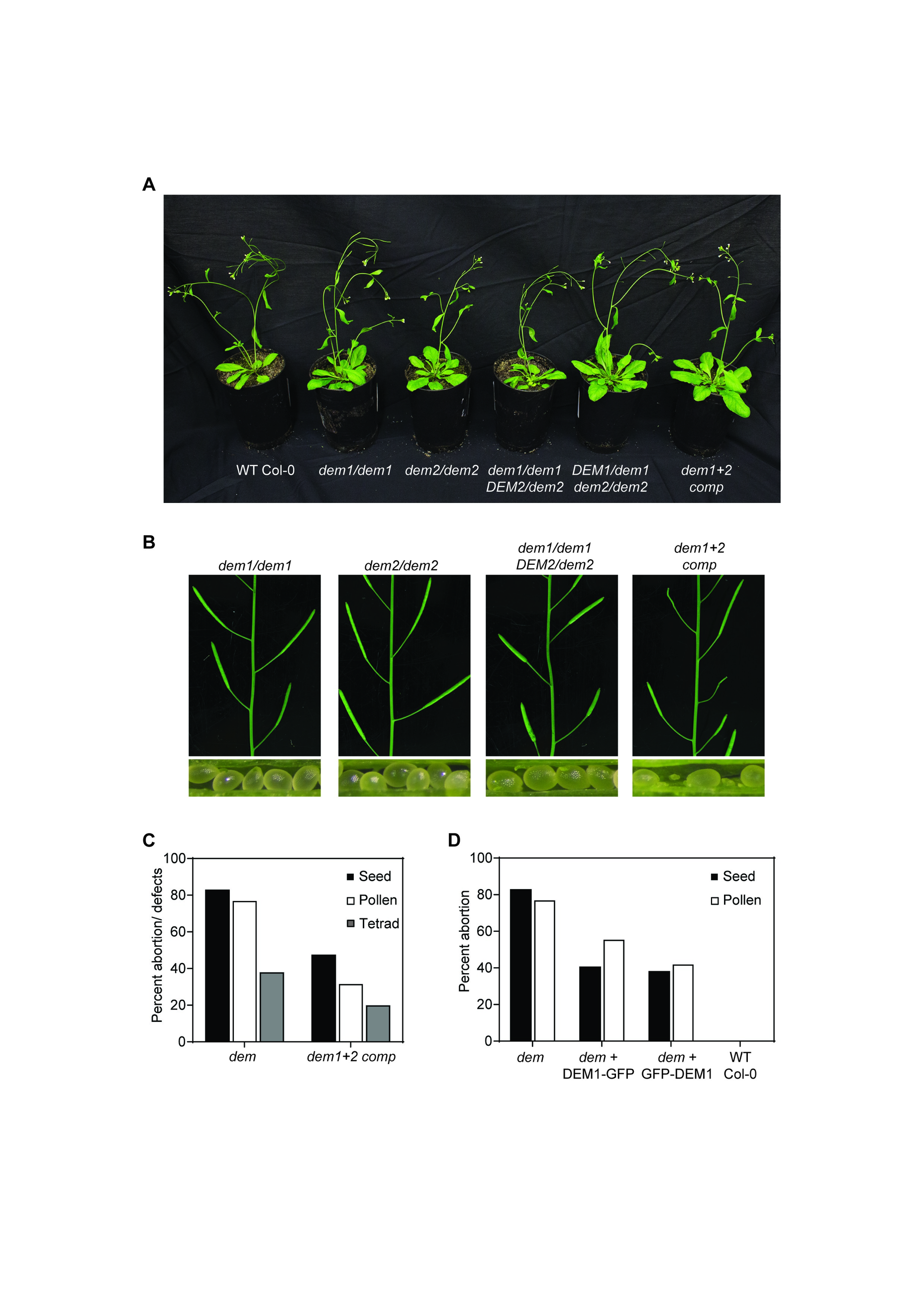

Supplement: S5 Fig — (A) Image showing 6-week old plants of wild type (WT) plants, dem1 and dem2 single mutants, dem1/dem1 DEM2/dem2, DEM1/dem1 dem2/dem2, and dem1 dem2 double mutant complemented with pDEM1:DEM1 transgene (dem1+2 comp) in Col-0 genetic background. (B) Normal seed production was observed in dem1 and dem2 single mutants and in dem1/dem1 DEM2/dem2 plants, but not in dem1/dem1 dem2/dem2 double mutant plants complemented with a pDEM1:DEM1 transgene (dem1+2 comp). (C) Percent ovule, pollen and tetrad defects in DEM1/dem1 dem2/dem2 plants (dem) and dem1/dem1 dem2/dem2 double mutant complemented with a pDEM1:DEM1 transgene (dem1+2 comp). At least 200 ovules, 500 pollen grains and 70 tetrads were assayed for each genotype. (D) Partial complementation of ovule and pollen abortion rates in DEM1/dem1 dem2/dem2 (dem) plants hemizygous for the pDEM1:GFP-DEM1 (GFP-DEM1) or pDEM1:DEM1-GFP (DEM1-GFP) transgene. Non-transgenic DEM1/dem1 dem2/dem2 (dem) and wild-type (WT) plants were included as controls. At least 200 ovules and 700 pollen grains were assessed for each genotype. (TIF) [file pgen.1009561.s005.tif]

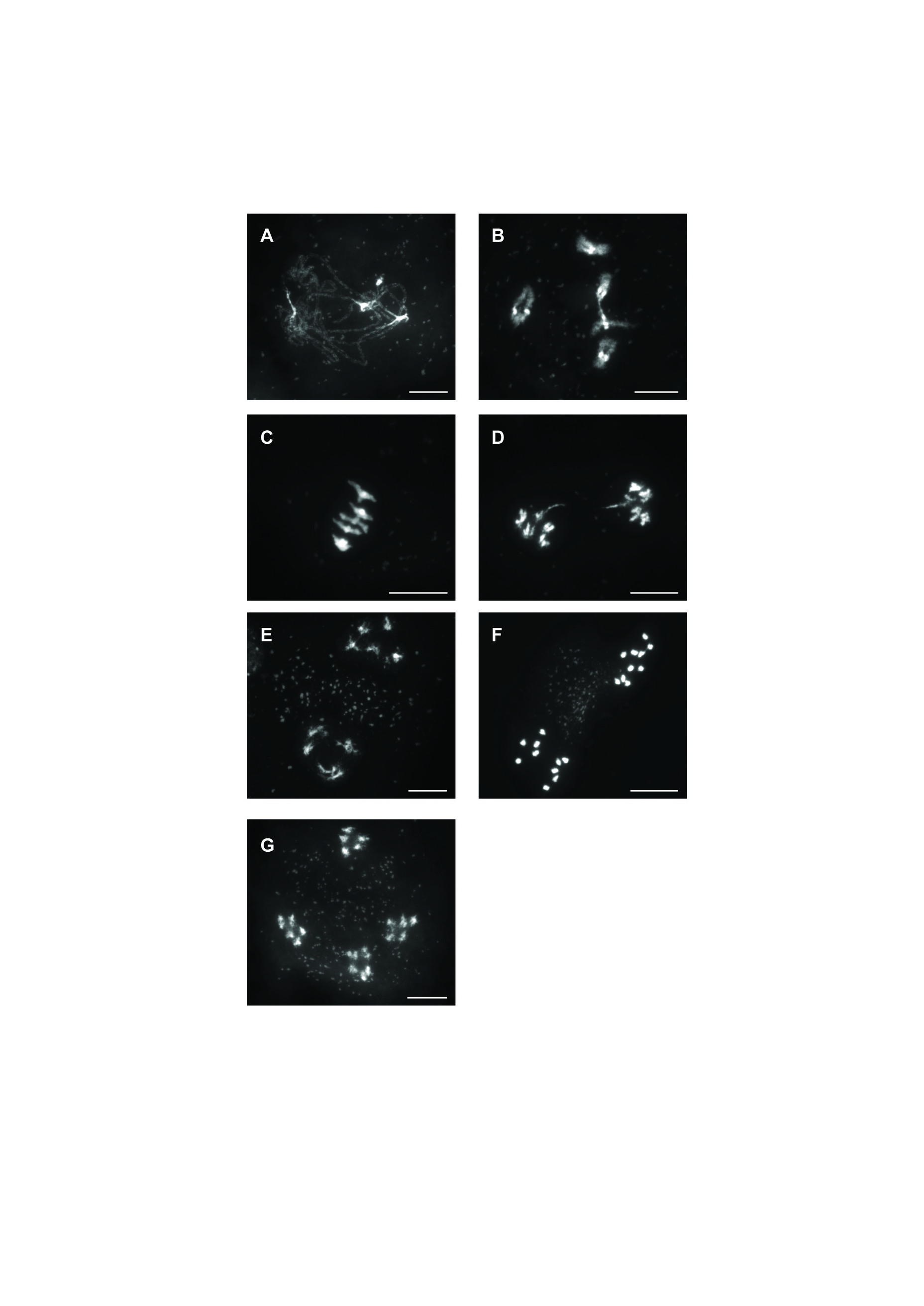

Supplement: S6 Fig — (A) Pachytene. (B) Diakinesis. (C) Metaphase I. (D) Anaphase I. (E) Telophase I. (F) Anaphase II. (G) Early telophase II. Bar = 10μm. (TIF) [file pgen.1009561.s006.tif]

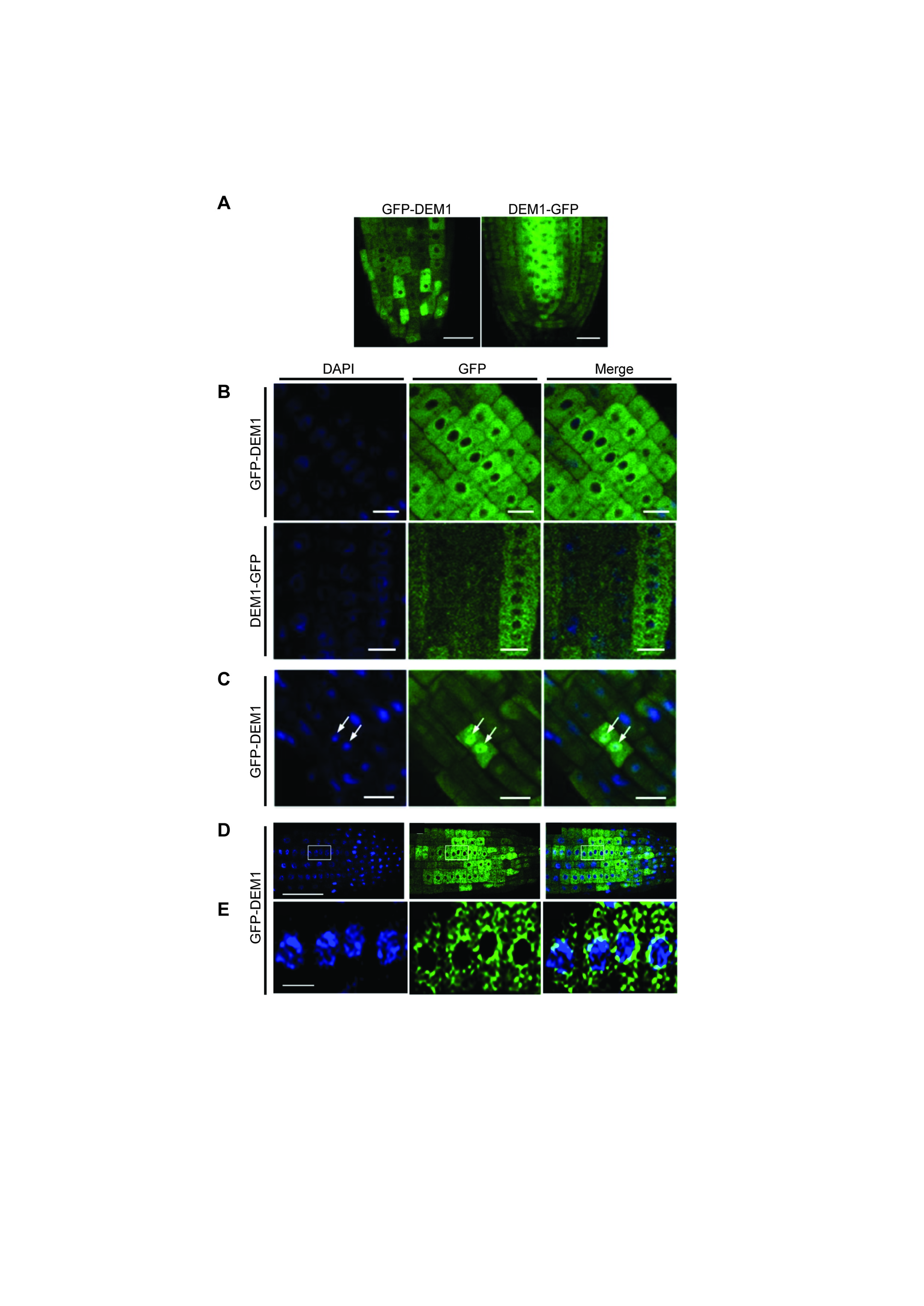

Supplement: S7 Fig — (A) Root tips of transgenic seedlings expressing GFP-DEM1 (left) or DEM1-GFP (right); bar = 20 μm. (B) DAPI-stained root tip cells expressing GFP-DEM1 or DEM1-GFP showing cytoplasmic and nuclear envelope localization; bar = 10 μm. (C) Localisation of GFP-DEM1 and DAPI in daughter cells of a root tip epidermal cell that has just divided. (D-E) Root tip expressing GFP:DEM1. (E) Higher magnification of sub-cellular localization of GFP:DEM1 in root tips corresponding to inset in (D), showing expression of GFP:DEM1 relative to the nucleus. Bar represents 50 μm for panels (A-D), and 10 μm for panels (E). Left-hand side, centre and right-hand side panels correspond to DAPI-stained, GFP and merged DAPI/GFP images, respectively (B-E). Images are representative of at least five independent transgenic lines for each transgene. (TIF) [file pgen.1009561.s007.tif]

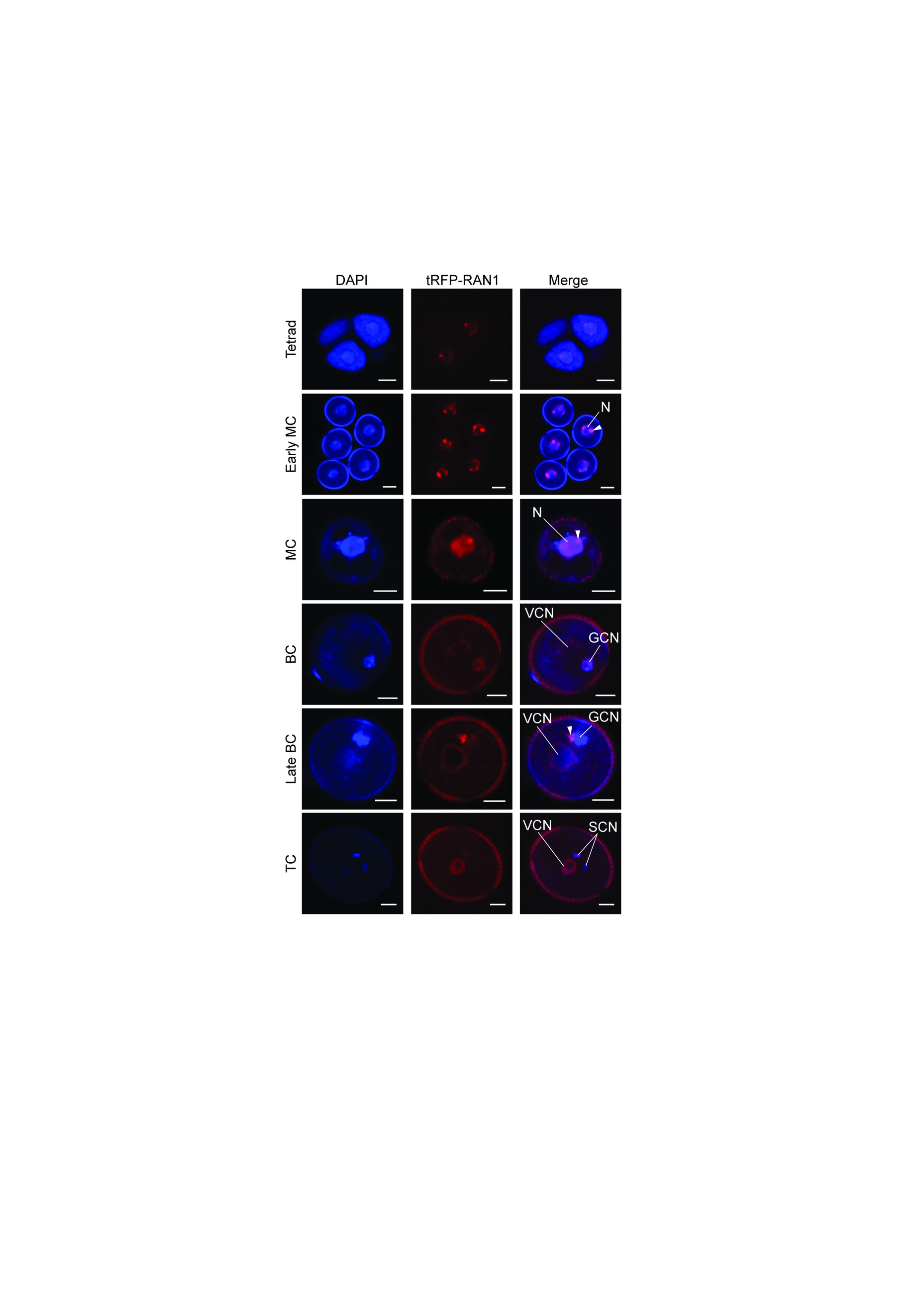

Supplement: S8 Fig — DAPI staining and localization of tRFP-RAN1 at various stages of microgametophyte development. In tetrads and free monocellular microspores (MC), tRFP-RAN1 predominantly co-localized with DAPI in the nucleus (N), but was concentrated in peripheral nuclear foci, particularly in early MC microgametophytes (white arrowhead). In early to mid-stage bicellular (BC) pollen, tRFP-RAN1 was mainly located in the generative cell nucleus (GCN) and weak expression was detected in the vegetative cell nucleus (VCN). In late BC pollen, tRFP-RAN1 was concentrated in extranuclear foci adjacent to the generative cell nucleus (white arrowhead; GCN), and a weak signal was detected in the vegetative cell nucleus. In tricellular (TC) pollen, tRFP-RAN1 was predominantly located in the vegetative cell nucleus (VCN). Bar = 5 μm. (TIF) [file pgen.1009561.s008.tif]

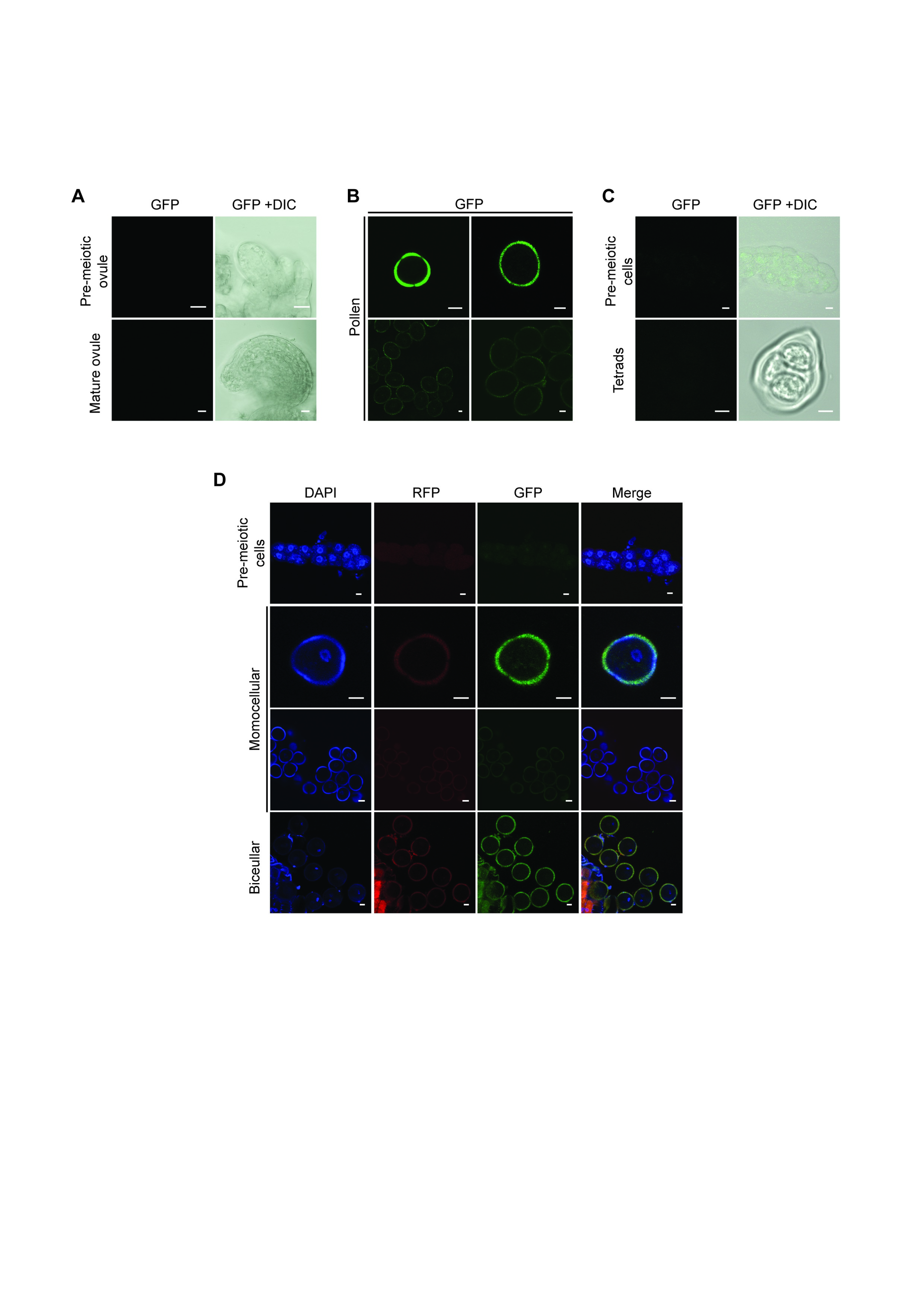

Supplement: S9 Fig — (A) GFP auto-fluorescence was not detected in pre-meiotic and mature ovules. (B) GFP auto-fluorescence was detected in the pollen coat, but not inside pollen grains. (C) GFP auto-fluorescence was not detected in pre-meiotic cells or tetrads. (D) RFP and GFP auto-fluorescence was not detected in the DAPI stained nuclei of pre-meiotic cells, monocellular and bicellular pollen. Auto-fluorescence of RFP and particularly GFP was detected in the pollen coat of monocellular and bicellular pollen. Bar = 5 μm. (TIF) [file pgen.1009561.s009.tif]

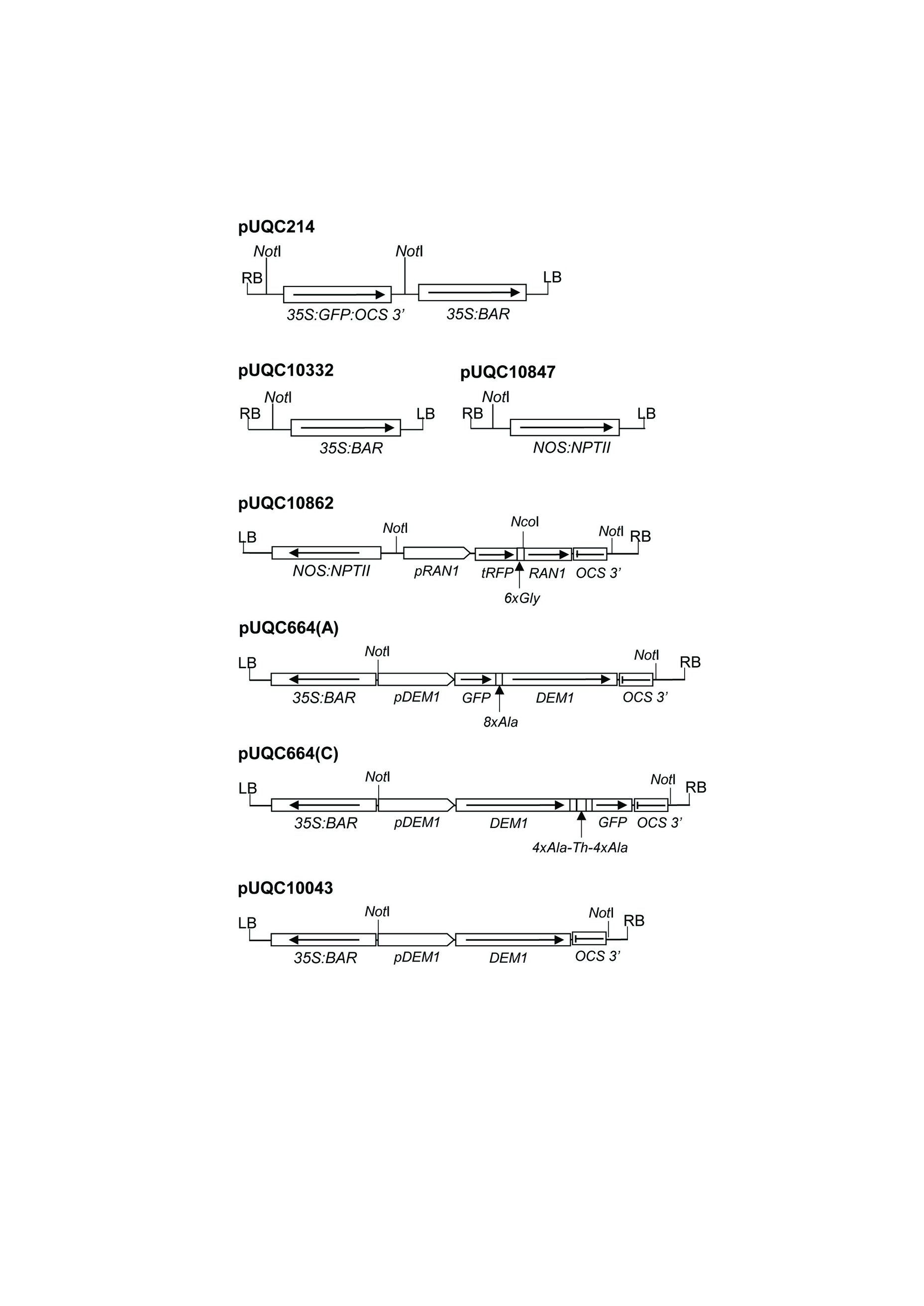

Supplement: S10 Fig — T-DNA binary vectors described in this study are all derived from the binary vectors pUQC477 and pUQC214 [71]. 35S represents the Cauliflower Mosaic Virus 35S promoter. The vector pUQC10332 is a derivative of pUQC214 with the 35S:GFP transgene removed, and it has a single NotI site upstream of the BAR selectable marker for inserting additional transgenes. pUQC10847 was constructed by removing the 35S:BAR sequence from pUQC477 [71], leaving a NPTII gene driven by the Agrobacterium NOPALINE SYNTHASE promoter (NOS) as a kanamycin selectable marker linked to a NotI site (for cloning in additional transgenes). OCS 3’, OCTOPINE SYNTHASE 3’ terminator. (TIF) [file pgen.1009561.s010.tif]
