# Supplementary material for: DEFECTIVE EMBRYO AND MERISTEMS genes are required for cell division and gamete viability in Arabidopsis
Source: PLoS Genet. 2021 May 17;17(5):e1009561. doi: 10.1371/journal.pgen.1009561 (PMC8158957; doi:10.1371/journal.pgen.1009561)
Supplement: S1 Table — (DOCX) [file pgen.1009561.s011.docx]

**S1 Table. Prediction of N-myristoylation (MYR) sites in DEM-like sequences in plants.**

MYR sites were predicted using the Myristoylator tool available from ExPASy (https://web.expasy.org/myristoylator/). The prediction of a MYR site and confidence are provided, along with the name of the organism, protein name and accession number. A score close to one indicates a high confidence for myristoylation.

| **Organism** | **Protein name** | **Protein accession** | **N-myristoylation** | **Confidence** |
| --- | --- | --- | --- | --- |
| *Volvox carteri* | hypothetical protein | XP_002954504.1 | No | - |
| *Volvox carteri* | hypothetical protein | XP_002954568.1 | Yes | 0.99 |
| *Chlamydomonas reinhardtii* | VID72-domain protein | XP_001697908.1 | Yes | 0.94 |
| *Micromonas commoda* | predicted protein, partial | XP_002500738.1 | Yes | 0.99 |
| *Klebsormidium flaccidum* | Vid27-related protein | GAQ90239.1 | Yes | 0.99 |
| *Physcomitrella patens* | predicted protein | XP_001767985.1 | Yes | 0.99 |
| *Physcomitrella patens* | predicted protein | XP_001785111.1 | Yes | 0.99 |
| *Physcomitrella patens* | predicted protein | XP_001782063.1 | No | - |
| *Picea abies* | VID27 cytoplasmic protein | MA_12899g0010 | Yes | 0.99 |
| *Picea abies* | VID27 cytoplasmic protein | MA_07580g0020 | Yes | 0.99 |
| *Picea abies* | VID27 cytoplasmic protein | MA_17978g0010 | Yes | 0.99 |
| *Solanum lycopersicum* | DEM protein | NP_001234563.1 | Yes | 0.99 |
| *Solanum lycopersicum* | DEM2 protein | NP_001234399.2 | Yes | 0.99 |
| *Arabidopsis thaliana* | Vid27-related protein | NP_195066.1 | Yes | 0.99 |
| *Arabidopsis thaliana* | Vid27-related protein | NP_188555.1 | Yes | 0.99 |
| *Medicago truncatula* | VID27-like protein | XP_003603039.1 | Yes | 0.99 |
| *Medicago truncatula* | VID27-like protein | XP_003607985.1 | Yes | 0.99 |
| *Brachypodium distachyon* | protein CYPRO4-like | XP_003570276.1 | Yes | 0.99 |
| *Brachypodium distachyon* | protein CYPRO4-like | XP_003570572.1 | Yes | 0.99 |
| *Orzya sativa* | PREDICTED: protein CYPRO4 | XP_015622974.1 | Yes | 0.99 |
| *Oryza sativa* | PREDICTED: protein CYPRO4 | XP_015626275.1 | Yes | 0.99 |
| *Sorgum bicolor* | hypothetical protein | XP_002452958.1 | Yes | 0.99 |
| *Sorgum bicolor* | hypothetical protein | XP_002453398.1 | Yes | 0.99 |
| *Zea mays* | protein CYPRO4 | NP_001152277.1 | No | - |
| *Zea mays* | protein CYPRO4 | NP_001140396.1 | No | - |
| *Marchantia polymorpha* | hypothetical protein | OAE35044.1 | Yes | 0.99 |
| *Selaginella moellendorffii* | hypothetical protein | XP_002980928.1 | No | - |
| *Selaginella moellendorffii* | hypothetical protein | XP_002989333.1 | No | - |
