# Supplementary material for: DEFECTIVE EMBRYO AND MERISTEMS genes are required for cell division and gamete viability in Arabidopsis
Source: PLoS Genet. 2021 May 17;17(5):e1009561. doi: 10.1371/journal.pgen.1009561 (PMC8158957; doi:10.1371/journal.pgen.1009561)
Supplement: S2 Table — (DOCX) [file pgen.1009561.s012.docx]

**S2 Table. Segregation analysis of wild-type *DEM* and mutant *dem* alleles in F_2_ progeny derived from crossing homozygous *dem1* and homozygous *dem2* single mutants.**

*N*, number of progeny scored. *P*, probability of χ2 for a chi-square distribution with four degrees of freedom. The expected segregation of genotypes assuming equal transmission of alleles is listed in brackets.

| **F_1_ plant** | ***N*** | **Segregation of genotypes in F_2_ progeny** | | | | | ***P**** |
| --- | --- | --- | --- | --- | --- | --- | --- |
|  |  | *dem1/dem1 dem2/dem2* | *DEM1/dem1 dem2/dem2* | *dem1/dem1 DEM2/dem2* | *DEM1/DEM1*  *DEM2/DEM2* | Other |  |
| *DEM1/dem1*  *DEM2/dem2*  (Ws-0) | 23 | 0  (1.43) | 1  (2.88) | 0  (2.88) | 5  (1.43) | 17  (14.37) | < 0.05 |
| *DEM1/dem1*  *DEM2/dem2*  (Col-0) | 40 | 0  (2.5) | 2  (5) | 1  (5) | 7  (2.5) | 30  (25) | < 0.05 |

**P* values below 0.05 were deemed as statistically significant.
