# Supplementary material for: DEFECTIVE EMBRYO AND MERISTEMS genes are required for cell division and gamete viability in Arabidopsis
Source: PLoS Genet. 2021 May 17;17(5):e1009561. doi: 10.1371/journal.pgen.1009561 (PMC8158957; doi:10.1371/journal.pgen.1009561)
Supplement: S3 Table — (DOCX) [file pgen.1009561.s013.docx]

**S3 Table. Segregation analysis of wild-type *DEM* and mutant *dem* alleles in progeny produced by self-fertilization of plants with one functional allele of *DEM*.**

*N*, number of progeny scored. The expected segregation of genotypes assuming equal transmission of wild-type *DEM* and mutant *dem* alleles is listed in brackets. *P*, probability of χ^2^ for a χ^2^ distribution with 2 degrees of freedom and an expected segregation ratio in the progeny of 1:2:1 (*dem1/dem1*

*dem2/dem2* : *DEM1/dem1 dem2/dem2 or dem1/dem1 DEM2/dem2* : *DEM1/DEM1 dem2/dem2 or dem1/dem1 DEM2/DEM2*)*. dem1/dem1 DEM2/dem2* plants in ecotype Ws-0 were not recovered*.*

| **Parent genotype** | ***N*** | **Segregation of genotypes in progeny of self-fertilized parent genotype** | | | ***P*** |
| --- | --- | --- | --- | --- | --- |
|  |  | *dem1/dem1*  *dem2/dem2* | *DEM1/dem1*  *dem2/dem2* or *dem1/dem1*  *DEM2/dem2* | *DEM1/DEM1*  *dem2/dem2* or *dem1/dem1 DEM2/DEM2* |  |
| *DEM1/dem1 dem2/dem2*  (Ws-0) | 17 | 0  (4.25) | 0  (8.5) | 17  (4.25) | <0.05 |
| *DEM1/dem1 dem2/dem2*  (Col-0) | 34 | 0  (8.5) | 11  (17) | 23  (8.5) | <0.05 |
| *dem1/dem1 DEM2/dem2*  (Col-0) | 21 | 0  (5.25) | 4  (10.5) | 17  (5.25) | <0.05 |
