# Supplementary material for: DEFECTIVE EMBRYO AND MERISTEMS genes are required for cell division and gamete viability in Arabidopsis
Source: PLoS Genet. 2021 May 17;17(5):e1009561. doi: 10.1371/journal.pgen.1009561 (PMC8158957; doi:10.1371/journal.pgen.1009561)
Supplement: S4 Table — (DOCX) [file pgen.1009561.s014.docx]

**S4 Table. RAN1 interacts with *Arabidopsis* DEM2 in the yeast two-hybrid system.**

Growth of six interacting preys on QDO SD media was rated as very strong (+++), moderate (++) or weak (+). The identity of inserts was identified as RAN1 by comparison of sequences to the NCBI database of *Arabidopsis* Expressed Sequence Tagged (EST) using the Blastn program. Growth test results showed there was a moderate to strong interaction between RAN1 and DEM2.

| **Bait** | **Prey sequence identity** | **Prey number** | **Prey insert size (bp)** | **Prey NCBI accession number** | **Prey locus number** | **Growth tests** | |
| --- | --- | --- | --- | --- | --- | --- | --- |
|  |  |  |  |  |  | **DEM1/2 bait** | **Unrelated baits**^b^ |
| DEM2 | RAN1 | 1, 4 and 5 | 467 ^a^ | X97379 | At5g20010 | ++ | - |
| DEM2 | RAN1 | 2, 3 and 50 | 751 ^a^ | X97379 | At5g20010 | +++ | - |

^a^ The full length of RAN1 mRNA is 1519 bp. ^b^ Empty bait vector and bait vector with the lamin C insert.
