# Supplementary material for: DEFECTIVE EMBRYO AND MERISTEMS genes are required for cell division and gamete viability in Arabidopsis
Source: PLoS Genet. 2021 May 17;17(5):e1009561. doi: 10.1371/journal.pgen.1009561 (PMC8158957; doi:10.1371/journal.pgen.1009561)
Supplement: S5 Table — (DOCX) [file pgen.1009561.s015.docx]

**S5 Table. Partial complementation of distorted segregation ratios by the *GFP-DEM1* and *DEM1-GFP* transgenes in *Arabidopsis* ecotype Col-0.**

*DEM1/dem1 dem2/dem2* plants carrying the *GFP-tagged DEM1* transgene were selfed and the progeny were PCR genotyped for the endogenous *dem1* and *dem2* alleles. *N*, number of progeny scored; *P*, probability for a chi-square distribution with one degree of freedom and an expected segregation based on *DEM1/dem1 dem2/dem2* plants with no transgene (in brackets).

| **Parent genotype** | **Transgene** | ***N*** | **Segregation of genotypes in progeny of self-fertilized parent genotype** | | | ***P**** |
| --- | --- | --- | --- | --- | --- | --- |
|  |  |  | ***dem1/***  ***dem1***  ***dem2/ dem2*** | ***DEM1/dem1***  ***dem2/ dem2*** | ***DEM1/DEM1***  ***dem2/ dem2*** |  |
| *DEM1/dem1*  *dem2/dem2* | None | 34 | 0 | 11  (11) | 23  (23) | n/a |
| *DEM1/dem1*  *dem2/dem2* | *pDEM1:GFP-DEM1* | 37 | 0 | 24  (12) | 13  (25) | < 0.05 |
| *DEM1/dem1*  *dem2/dem2* | *pDEM1:DEM1‑GFP* | 109 | 0 | 50  (35.3) | 59  (73.7) | < 0.05 |

**P* values below 0.05 were deemed as statistically significant. n/a, not applicable.
