# Supplementary material for: DEFECTIVE EMBRYO AND MERISTEMS genes are required for cell division and gamete viability in Arabidopsis
Source: PLoS Genet. 2021 May 17;17(5):e1009561. doi: 10.1371/journal.pgen.1009561 (PMC8158957; doi:10.1371/journal.pgen.1009561)
Supplement: S6 Table — (DOCX) [file pgen.1009561.s016.docx]

**S6 Table. Partial complementation of defective *dem* pollen by GFP-tagged *DEM1* transgenes.**

Crosses were made between wild-type female plants and *DEM1/dem1 dem2/dem2* male plants (Col-0) carrying the *GFP-tagged DEM1* transgene. *N*, number of progeny scored.

| **Cross**  **(♀ x ♂)** | **Number of crosses attempted** | **Progeny**  **carrying transgene** | **Segregation of genotypes in progeny** | | ***N*** |
| --- | --- | --- | --- | --- | --- |
|  |  |  | ***dem1***  ***dem2*** | ***DEM1***  ***dem2*** |  |
| WT x *DEM1/dem1 dem2/dem2*  (no *GFP* transgene)^a^ | 5 | n/a | n/a | n/a | 0^b^ |
| WT x *DEM1/dem1 dem2/dem2*  carrying homozygous *pDEM1:GFP-DEM1* transgene^a^ | 1 | Yes | 3 | 10 | 13 |
| WT x *DEM1/dem1 dem2/dem2*  carrying hemizygous *pDEM1:GFP-DEM1* transgene^a^ | 2 | Yes | 0 | 6 | 6 |
|  |  | No | 0 | 5 | 5 |
| WT x *DEM1/dem1 dem2/dem2*  carrying hemizygous *pDEM1:DEM1‑GFP* transgene^a^ | 3 | Yes | 0 | 15 | 15 |
|  |  | No | 0 | 17 | 17 |

^a^ *DEM1/dem1 dem2/dem2* plants used as males in crosses either lacked the *pDEM1:DEM1‑GFP* and *pDEM1:DEM1‑GFP* transgene or were homozygous or hemizygous for either transgene. The zygosity of the transgene in the male parent was determined by genotyping the progeny of the cross for the *GFP-tagged DEM1* transgene. ^b^No progeny were obtained from five separate experiments involving multiple *DEM1/dem1 dem2/dem2* plants; n/a, not applicable due to male sterility of the *DEM1/dem1 dem2/dem2* mutant when used in a cross.
