# Supplementary material for: DEFECTIVE EMBRYO AND MERISTEMS genes are required for cell division and gamete viability in Arabidopsis
Source: PLoS Genet. 2021 May 17;17(5):e1009561. doi: 10.1371/journal.pgen.1009561 (PMC8158957; doi:10.1371/journal.pgen.1009561)
Supplement: S7 Table — (DOCX) [file pgen.1009561.s017.docx]

**S7 Table. Oligonucleotides used in this study.**

| **Oligonucleotide** | **Sequences**^a^ | **Description** |
| --- | --- | --- |
| DEM13´End-REV | GCAGTAGCATTTCTTCAGCCCTTCC | To genotype *dem1-1* |
| JL202 | CATTTTATAATAACGCTGCGGACATCTAC |  |
| DEM1-mid-5´-inner | GACCGATATGGGATGGTTCAGGATCT | To genotype *DEM1*  (Ws-0 ecotype) |
| DEM13´End-REV | GCAGTAGCATTTCTTCAGCCCTTCC |  |
| DEM2_5´_primer | AATCCCTAATCCTCCTCTAAATCCCTAAC | To genotype *dem2-1* |
| JL202 | CATTTTATAATAACGCTGCGGACATCTAC |  |
| DEM2_5´_primer | AATCCCTAATCCTCCTCTAAATCCCTAAC | To genotype *DEM2*  (Ws-0 ecotype) |
| DEM2(ws)_zyg_REV | TCTGCACTCCACCATTAGCCACC |  |
| RP-DEM1-mod | CGACTTTCAAAATCCACCAT | To genotype *dem1-2* |
| LBb1.3_mod | GGCTTTTGGCGATTTCGGAAC |  |
| RP-DEM1-mod | CGACTTTCAAAATCCACCAT | To genotype *DEM1*  (Col-0 ecotype) |
| RT-PCR-DEM1-FWD | GGAAGAGAGGGAGAGGAAGAGGAAG |  |
| RP_SLK070099 | GTTCTTCACCCGCTCCAAGTCC | To genotype *dem2-2* |
| LBb1.3_mod | GGCTTTTGGCGATTTCGGAAC |  |
| LK_SLK070099 | AACAAGGGAAACCTAACTCCACTGG | To genotype *DEM2*  (Col-0 ecotype) |
| RP_SLK070099 | GTTCTTCACCCGCTCCAAGTCC |  |
| LP_DEM1 | GCCTAAATCTCCTTCATCATCACTCG | Large RNA northern and southern blot probes for  *DEM1 3'* |
| RP_DEM1 | CCGACTTTCAAAATCCACCATTCG |  |
| AtD5'-1 | *GGATCCATCGATGGTAC*CTTCTCAGAGTGGTGAAGA | Large RNA northern and southern blot probe for  *DEM1 5'* and *DEM2 5'* |
| AtD3’-2 | *GCCCTGGATCCGAATTC*CTAGTAACAAACTTCC | Large RNA northern and southern blot probe for  *DEM2 5'* |
| Atd3’-3 | *GCCCTGGATCCGAATTC*CTGCTTACAAAAACCGA | Large RNA northern and southern blot probe for  *DEM1 5'* |
| DEM1-qRT-PCR-F | TGGTCATTGCAACGCCTATG | *DEM1* qRT-PCR for various tissues, except floral buds |
| DEM1-qRT-PCR-R | ATAACTAGTGGTCCGGTCCATCAC | *DEM1* qRT-PCR for various tissues, except floral buds |
| DEM1 859-880 F | CCCCGTCTACTCAGAACTCAGC | *DEM1* qRT-PCR for wild-type and *dem* floral buds |
| RP-DEM1-mod | CGACTTTCAAAATCCACCAT | *DEM1* qRT-PCR for wild-type and *dem* floral buds |
| DEM2-qRT-PCR-F: | CCTTCTCCGGTAACAAATCGC- | *DEM2* qRT-PCR |
| DEM2-qRT-PCR-R: | AAAGTCGTTTTCCAGAGAGGCTG | *DEM2* qRT-PCR |
| beta-actin F: | AGTGGTCGTACAACCGGTATTGT | *β-actin* qRT-PCR |
| beta-actin-R2: | GATGGCATGGAGGAAGAGAGAAAC | *β-actin* qRT-PCR |
| beta-actin-R7: | GAGGAAGAGCATTCCCCTCGTA | *β-actin* qRT-PCR |
| beta-actin-R8: | GAGGATAGCATGTGGAACTGAGAA | *β-actin* qRT-PCR |

^a^Sequences in italics within an oligonucleotide do not match the target gene.
