# Supplementary material for: DEFECTIVE EMBRYO AND MERISTEMS genes are required for cell division and gamete viability in Arabidopsis
Source: PLoS Genet. 2021 May 17;17(5):e1009561. doi: 10.1371/journal.pgen.1009561 (PMC8158957; doi:10.1371/journal.pgen.1009561)
Supplement: S1 Text — (DOCX) [file pgen.1009561.s018.docx]

**S1 Text. Supplementary materials and methods.**

**N-myristoylation (MYR) prediction and pairwise sequence alignment**

To predict N-myristoylation sites, plants DEM-like sequences were submitted to the Myristoylator tool available from ExPASy (Bologna *et al.*, 2004). Pairwise sequence alignment of *Arabidopsis* DEM1 (NP_195066.1) and nuclear rim localization domain of VID27-like protein from S*chizosaccharomyces pombe* (BAA87237.1) was conducted using the LALIGN tool from EMBL-EBI (http://www.ebi.ac.uk/Tools/psa/lalign/). The default parameters used for pairwise alignment were: matrix, BLOSUM50; gap open penalty, -12; gap extend penalty, -2; and expectation threshold, 10.

**PCR genotyping of T-DNA insertion lines**

For PCR genotyping, DNA template was extracted as described previously (Edwards *et al.*, 1991). Oligonucleotide primers used for genotyping T-DNA insertions are detailed in S7 Table. To genotype the endogenous *DEM1* wild-type allele in *pDEM1:DEM1-GFP* and *pDEM1:GFP-DEM1* transgenic lines, an intron-specific primer (not recognizing transgene sequences) was used.

**Yeast two-hybrid assays**

The Matchmaker Library Construction and Screening kit (Clontech Laboratories Inc., California) was used to construct *Arabidopsis* cDNA libraries and screen them with bait vectors containing the *DEM1* and *DEM2* cDNAs. The 1947-base pair (bp) *DEM1* cDNA was amplified with the oligonucleotide primers 5´- TTTCCCGGGCATGGGTACTTCTCAG-3´ and 5´-CGTTGGATCGTCTGAAGAGGAGG-3´, and the 1938-bp *DEM2* cDNA was amplified with the oligonucleotides 5´- TTTCCCGGGTATGGGAGCATCTCACAGTCATGAAG-3´ and 5´-CAGAGGTTGATGTGAATAACGG-3´. Both 5´ oligonucleotides included *XmaI* sites to facilitate in-frame cloning into bait vectors. The PCR products were cloned into pGEM-T easy (Promega) to produce the constructs and pUQC23016 (*DEM1* cDNA) and pUQC2321 (*DEM2* cDNA). Both clones were selected so as to have the *PstI* site from the pGEM-T easy multi-cloning site at the 3´ end of the cDNAs, and these clones were sequenced to check integrity of the inserts. The *DEM2* cDNA was sub-cloned from pUQC2321 into pGBKT7 as an *XmaI-PstI* fragment, to create pUQC2333. Consistent difficulties were experienced in sub-cloning the *DEM1* cDNA from pUQC23016 into pGBKT7. It was instead cloned into another bait vector, pGBT9, as an *XmaI-PstI* fragment, to produce pUQC2311. PCR positive clones for both bait constructs were checked using test digests and sequencing to ensure the integrity and correct reading frame of the inserts. Total RNA was extracted from a mixture of mature silique, floral bud, young leaf and mature leaf tissues, and cDNA prey libraries were made according to Clontech’s instructions. The two-hybrid library screens were carried out by mating bait-carrying Y187 lines to the library-carrying AH109 lines, with the resulting diploids plated onto quadruple dropout (QDO) SD media to select for activation of the *ADE2* and *HIS3* markers, according to Clontech’s instructions. To ensure that the prey plasmids identified were not false positives, further tests were conducted as described by Bartel *et al.* (Bartel *et al.*, 1993; Bartel and Fields, 1995).

**Construction of binary vectors for stable expression of GFP and tagRFP fusion proteins in *Arabidopsis***

T-DNA binary vectors described in this study are all derived from the binary vectors pUQC477 and pUQC214 (Brosnan *et al.*, 2007), and are shown in S10 Fig.

All PCR reactions for cloning were performed using Expand High Fidelity PCR system (Roche). PCR products were cloned into pGEM-T Easy vector and sequenced to confirm authenticity. To clone the *DEM1* promoter (*pDEM1*), a region 1228 bp upstream of the *DEM1* start codon was amplified using genomic DNA template (ecotype Col-0) and oligonucleotide primers incorporating 5΄ SacI and a 3΄ NcoI ends. The *DEM1* promoter region was inserted into the multiple cloning site of a pUC vector containing Green Fluorescence Protein (GFP S65T; GenBank accession no. U43284) followed by a 3´ *octopine synthase (ocs)* terminator from *Agrobacterium tumefaciens* (Brosnan *et al.*, 2007). The *pDEM1:GFP:ocs* intermediate vector was designated as pUQC660 and contained a single *NcoI* site between the *DEM1* promoter and the start codon of the *GFP* coding sequence. To construct a *pDEM1:DEM1-GFP:ocs* intermediate vector, the *DEM1* coding sequence was amplified using cDNA template (ecotype Col-0) and primers introducing an *NcoI* site at the 5΄ and 3΄ ends. The 3΄ primer contained an additional coding sequence for a linker containing 4 alanine residues, a thrombin cleavage site, followed by another four alanine residues and the *pDEM1:DEM1-GFP:ocs* intermediate vector was designated pUQC661(B).

To construct a *pDEM1:GFP-DEM1:ocs* intermediate vector, pUQC654 (S10 Fig) had an unwanted *NcoI* site downstream of the *DEM1* coding sequence first removed. The *GFP* sequence from pUQC660 was excised with *NcoI* and *BsrGI* and inserted into the *NcoI* site of pUQC654 in combination with an oligo-pair containing 5΄ *BsrGI* and 3΄ *NcoI* cohesive ends and the coding sequence for an eight alanine linker. The *GFP:DEM1* sequence was then removed by *NcoI* and *BamHI* partial digestion and inserted back into pUQC660 to construct the *pDEM1:GFP-DEM1:ocs* vector designated as pUQC661. The *GFP-DEM1* and *DEM1-GFP* transgenes from pUQC661 and pUQC661(B) were then cloned into derivatives of the binary vector pUQC214 to form the T-DNA vectors pUQC664(A) and pUQC664(C), respectively. These vectors carry *35S:BAR:nos* as a plant selectable marker and the T-DNA maps are shown in S10 Fig.

To construct the *pDEM1:DEM1:ocs* transgene, *NcoI* was used to remove the *GFP* coding sequence from pUQC661 to create pUQC10042. The *pDEM1:DEM1:ocs* *NotI* fragment was then excised from pUQC10042 and cloned into the binary vector backbone of pUQC10332 to create pUQC10043 (S10 Fig).

To construct the *pRAN1:tRFP-RAN1* binary vector, the 685 bp promoter region (*pRAN1*) and the *RAN1* coding sequence were synthesised by GeneArt (Invitrogen; pUQC10849). An *NcoI* site was introduced into pUQC10849 at the start codon of the *RAN1* coding sequence by conversion of a nucleotide 2 bp upstream of the start codon from an A to C. An internal *NcoI* site in the *RAN1* coding sequence was also removed by a substitution of nucleotide 255 in the coding sequence from T to C, introducing a silent mutation. The *pRAN1:RAN1* sequence was cloned into a derivative of pUQC660 to construct a *pRAN1:RAN1:ocs* 3' intermediate pUC vector, which was designated pUQC10848. The *tagRFP* coding sequence without a stop codon was amplified using oligonucleotide primers that introduced 5΄ and 3΄ *NcoI* sites, using the pUC vector pSITE-6C1 as a template. This *tagRFP NcoI* fragment was inserted into the *NcoI* site of pUQC10848 to produce the pUC construct pUQC10851 carrying the *pRAN1:tagRFP:RAN1:ocs* transgene. The 3΄ primer used to amplify the *tagRFP* coding sequence also incorporated six glycines that served as a flexible linker between the *tagRFP* and *RAN1* coding sequences in pUQC10851. The *pRAN1:tagRFP:RAN1:ocs* transgene was then cloned as a *NotI* fragment into pUQC10847 to create pUQC10862 (S10 Fig).

Further details of construction of these binary vectors can be obtained from the authors.

**Stable expression of GFP and tagRFP fusion proteins in *Arabidopsis***

Binary vectors *pDEM1:GFP-DEM1, pDEM1:DEM1-GFP* or *pRAN1:tRFP-RAN1* (S10 Fig) were introduced into *Agrobacterium tumefaciens* strain GV3101::MP90, and the floral dip transformation protocol (Clough and Bent, 1998) was used to produce transgenic *Arabidopsis* lines expressing DEM1-GFP and GFP-DEM1. To generate transgenic lines co-expressing *pRAN1:tRFP-RAN1* with *pDEM1:DEM1-GFP* or *pDEM1:GFP-DEM1*, plants were co-transformed and selected on MS media with 50 µg/ml phosphinothricin (PPT) and 75 µg/ml kanamycin. For confocal microscopy and localization studies (see below), T_1_ or T_2_ plants were analyzed.

**Expression and purification of recombinant proteins**

The *Arabidopsis RAN1* coding region (At5g20010) was expressed as a GST-tagged fusion protein. *RAN1* was amplified from *Arabidopsis* cDNA using primers that introduced 5΄ *BamHI* and 3΄ *EcoRI* sites and subsequently cloned into pGEX-KG (Guan and Dixon, 1991) to form pUQC641.

The plasmid pUQC641 was introduced into *E. coli* strain BL21(DE3) (Novagen) carrying the plasmid pGTf2 (Takara Bio) coding for a set of chaperones (groES-groEL-tig). Cells were grown in 2 L of terrific broth medium containing 200 μg/ml ampicillin and 35 μg/ml chloramphenicol at 37ºC to a cell density of 1 to 2 *A*_600_ units. Cells were cooled down on ice and 1 mM of isopropyl-1-thio-β-D-galactopyranoside (IPTG) and 10 ng/ml of tetracycline were added to induce expression of recombinant proteins and chaperones, respectively. After incubation on a shaker (160 rpm) for 16-20 hours at 18ºC, cells were harvested by centrifugation and the pellet was frozen at -20ºC.

All purification steps were carried out at 5ºC. The pellets were thawed and resuspended in 50 ml of appropriate buffer (PBS for GST:AtRAN1 or 50 mM Tris pH 8, 10 mM imidazole for His_6_-DEM1) containing 1x FastBreak Cell Lysis (Promega), 1 mM DTT, 1 μg/ml of protease inhibitors (aprotine, leupeptin and pepstatin), 1 mM PMSF and 100 units of DNase. The homogenates were centrifuged at 25,000 x *g* for 1 h and the cleared supernatants were subject to chromatography. The supernatants containing GST-RAN1 were subjected to chromatography on Glutathione-Agarose columns following the manufacturer’s instruction (Scientifix).

**Protein-binding assays**

To pull-down DEM1-GFP and GFP-DEM1 from plant extracts using GST-tagged RAN1, transgenic tissues (0.5-1g) expressing GFP-tagged proteins were homogenized by snap-freezing in liquid nitrogen, crushing with mortar and pestle and adding 3 ml extraction buffer (20 mM Tris, pH 8.0, 150 mM NaCl, 2.5 mM EDTA, 2 mM dTT, 0.5% (v/v) NP-40 and protease inhibitor cocktail (Roche)). The plant extract was incubated with agitation at 4°C for 30 min and centrifuged at 10,000 x *g* for 15 min at 4°C to remove cellular debris. The supernatant were then mixed with 1 mg GST-RAN1 or 1 mg GST and incubated for 20 min at 4°C and a further 20 min with 200 µl of glutathione sepharose 4B beads. The beads were then washed with 6 x 1 ml protein extraction buffer. To remove purified protein and co-purified protein complexes, 50 µl of 2x loading buffer was added and incubated at 95°C for 2-5 min. Western detection of pulled-down proteins was carried out using mouse anti-GFP antibody (Roche) and Alexa Fluor 680 goat anti-mouse antibody (Invitrogen, Molecular Probes).

**References**

**Bartel, P., Chien, C.T., Sternglanz, R. and Fields, S.** (1993) Elimination of false positives that arise in using the two-hybrid system. *BioTechniques*, **14**, 920-924.

**Bartel, P.L. and Fields, S.** (1995) Analyzing protein-protein interactions using two-hybrid system. *Methods in Enzymology*, **254**, 241-263.

**Bologna, G., Yvon, C., Duvaud, S. and Veuthey, A.L.** (2004) N-Terminal myristoylation predictions by ensembles of neural networks. *Proteomics*, **4**, 1626-1632.

**Brosnan, C.A., Mitter, N., Christie, M., Smith, N.A., Waterhouse, P.M. and Carroll, B.J.** (2007) Nuclear gene silencing directs reception of long-distance mRNA silencing in *Arabidopsis*. *PNAS*, **104**, 14741-14746.

**Clough, S.J. and Bent, A.F.** (1998) Floral dip: a simplified method for Agrobacterium-mediated transformation of *Arabidopsis thaliana*. *Plant Journal*, **16**, 735-743.

**Edwards, K., Johnstone, C. and Thompson, C.** (1991) A simple and rapid method for the preparation of plant genomic DNA for PCR analysis. *Nucleic Acids Research*, **19**, 1349.

**Guan, K.L. and Dixon, J.E.** (1991) Eukaryotic proteins expressed in Escherichia coli: an improved thrombin cleavage and purification procedure of fusion proteins with glutathione S-transferase. *Analytical Biochemistry*, **192**, 262-267.
